# Targeting soluble tumor necrosis factor as a potential intervention to lower risk for late-onset Alzheimer’s disease associated with obesity, metabolic syndrome, and type 2 diabetes

**DOI:** 10.1186/s13195-019-0546-4

**Published:** 2019-12-31

**Authors:** Maria Elizabeth De Sousa Rodrigues, Madelyn C. Houser, Douglas I. Walker, Dean P. Jones, Jianjun Chang, Christopher J. Barnum, Malú G. Tansey

**Affiliations:** 1 Department of Physiology, School of Medicine at Emory University, 615 Michael Street, Atlanta, GA 30322-3110 USA; 2 Division of Pulmonary, Allergy and Critical Care Medicine, Emory University Emory, 615 Michael Street, Atlanta, GA 30322 USA; 3 Department of Environmental Medicine and Public Health, Icahn School of Medicine at Mount Sinai, New York, NY 10003 USA; 4 Department of Neuroscience, Center for Translational Research in Neurodegenerative Disease, University of Florida College of Medicine, McKnight Brain Institute, Gainesville, FL USA

**Keywords:** Insulin, Soluble tumor necrosis factor, Metabolic inflammation, Neuroactive metabolites, Lipocalin-2, Purines, Proteoglycans, Gut, Liver, Metabolomics

## Abstract

**Background:**

Insulin impairment and inflammation are two features common to type 2 diabetes and Alzheimer’s disease; however, the molecular and signaling interactions underlying this relationship are not well understood. Mounting evidence point to the associations between the disruption of metabolite processing in insulin impairment and neurodegenerative conditions such as Alzheimer’s. Although the brain depends partially on metabolites processed in the periphery, to date, little is known about how soluble tumor necrosis factor signaling (solTNF) impacts integrated peripheral immune and metabolic feedback signals in states of energy overload and insulin insensitivity.

**Methods:**

C57Bl/6J mice were fed a high-fat high-carbohydrate diet (HFHC) for 14 weeks. The brain-permeant biologic XPro1595® was used to block solTNF-dependent pathways. Metabolic and immune alterations were evaluated in the gut, liver, and brain. Behavioral tests were performed. Untargeted metabolomics was carried out in the plasma and liver.

**Results:**

HFHC diet promotes central insulin impairment and dysregulation of immune-modulatory gene expressed in the brain. Alteration of metabolites associated with type 2 diabetes and Alzheimer’s such as butanoate, glutamate, biopterin, branched-chain amino acids, purines, and proteoglycan metabolism was observed in HFHC-fed mice. solTNF inhibition ameliorates hepatic metabolic disturbances and hepatic and intestinal lipocalin-2 levels, and decreases insulin impairment in the brain and behavioral deficits associated with HFHC diet.

**Conclusions:**

Our novel findings suggest that HFHC diet impacts central insulin signaling and immune-metabolic interactions in a solTNF-dependent manner to increase the risk for neurodegenerative conditions. Our novel findings indicate that selective solTNF neutralization can ameliorate peripheral and central diet-induced insulin impairment and identify lipocalin-2 as a potential target for therapeutic intervention to target inflammation and insulin disturbances in obesogenic environments. Collectively, our findings identify solTNF as a potential target for therapeutic intervention in inflammatory states and insulin disturbances in obesogenic environments to lower risk for AD.

## Background

Insulin resistance (IR) affects an increasingly large population globally, and despite decades of intense research efforts, type 2 diabetes (T2D) remains an important public health problem throughout the world [1]. Central IR disrupts memory and cognition and promotes disturbance in metabolic and inflammatory responses [2]. Although IR and systemic inflammation have been identified as risk factors for Alzheimer’s disease (AD), the molecular and signaling mechanism underlying this relationship are not well understood. In the states of over-nutrition, intestinal microbiota-derived products and circulating food metabolites from intestinal and liver interactions can disrupt the regulation of insulin activities and immune balance.

A wealth of the literature suggests that elevated tumor necrosis factor (TNF) exerts central and peripheral effects on metabolic and immune pathways and contributes to IR and AD [3–7]. TNF is upregulated in the presence of obesity and impacts the expression of other multiple inflammatory factors such as IL-6 and LCN2 that promote, exacerbate, and sustain chronic systemic inflammation and insulin impairment [6, 8]. TNF is synthesized as a type I transmembrane protein (tmTNF) that is biologically active in innate immune defense against infection and in myelination [7]. Once cleaved to a soluble (solTNF) form, it is able to mediate inflammatory processes [9, 10]. The overlap and synergistic effects of TNF on metabolic pathways can impact insulin sensitivity and diabetes comorbidities [4, 5].

Additionally, this pleiotropic cytokine is implicated in the central and hepatic interactions that control glucose metabolism and insulin functions [11, 12]. Therefore, maladaptive processes implicated in hepatic steatosis and liver inflammation are associated with the central effects of TNF on insulin dysregulation [3, 13]. In addition to its tissue direct effects on IR, TNF regulates pro-inflammatory markers, such as IL-6 and lipocalin-2 (LCN2) that are implicated in the pathogenesis of hepatic steatosis and T2D onset and progression [14–16]. Centrally, LCN2 promotes chemokine production in the brain in response to inflammatory insults and regulates glial cells activity and neuroinflammatory and neurodegenerative processes [16]. Some immune-related effects of LCN2 include its deleterious effects in aging insulin insensitivity [14].

Despite the evidence linking anti-TNF strategies with the improvement of insulin sensitivity, it is not known how the selective neutralization of solTNF signaling can impact the deleterious metabolic-immune interactions present in obesity that affects IR [17, 18]. The hypothesis tested here is that solTNF drives metabolic and inflammatory changes in the gut-liver axis that contributes to insulin impairment and the systemic metabolic dysregulation that increases the risk for AD. We previously demonstrated that the brain-permeant dominant-negative solTNF-selective inhibitor XPro1595® decreases beta-amyloid plaque load in the 5XFAD animal model of AD-like pathology [19]. In the current study, we used this agent to assess the effect of inhibition of central and peripheral solTNF activity in diet-induced insulin impairment. This biologic inhibits solTNF signaling by forming inactive heterotrimers with native solTNF to sequester it away from interacting with TNF receptors [20]. Therefore, this approach leaves the host defense and the neuroprotective transmembrane TNF signal intact [7, 21]. Amyloid deposition was not assessed in our animal model because our previous studies and other groups demonstrate that C57BL/6J mice may present amyloid aggregates in older age (15 months or more) as a result of the aging process, and HFHC diet effects on amyloid deposition in our non-transgenic mice were not anticipated [22]. The experimental design of this study explores a more physiologic approach to assess the metabolic and immune risks for AD associated with an obesogenic environment.

## Materials and methods

### Animals and diet intervention

C57Bl/6 male mice (*n* = 50, 6 weeks old, The Jackson Laboratory, Bar Harbor) were singly housed in a colony room (22–23 °C with a 12/12-h light-dark cycle). After 7 days of acclimation, mice received drinking water and standard chow diet (4% fat diet 7001, Envigo) or high-fat high-carbohydrate diet (HFHC) (42% kcal from fat, TD.88137, Envigo) plus 30% (w/v) fructose solution (F012, Sigma-Aldrich) available ad libitum for 14 weeks. Food and drink consumptions were measured twice a week. Mice were weighed weekly (Protocol # DAR-2003358-ENTRPR-N).

### Soluble TNF neutralization

**Fig. 1:**
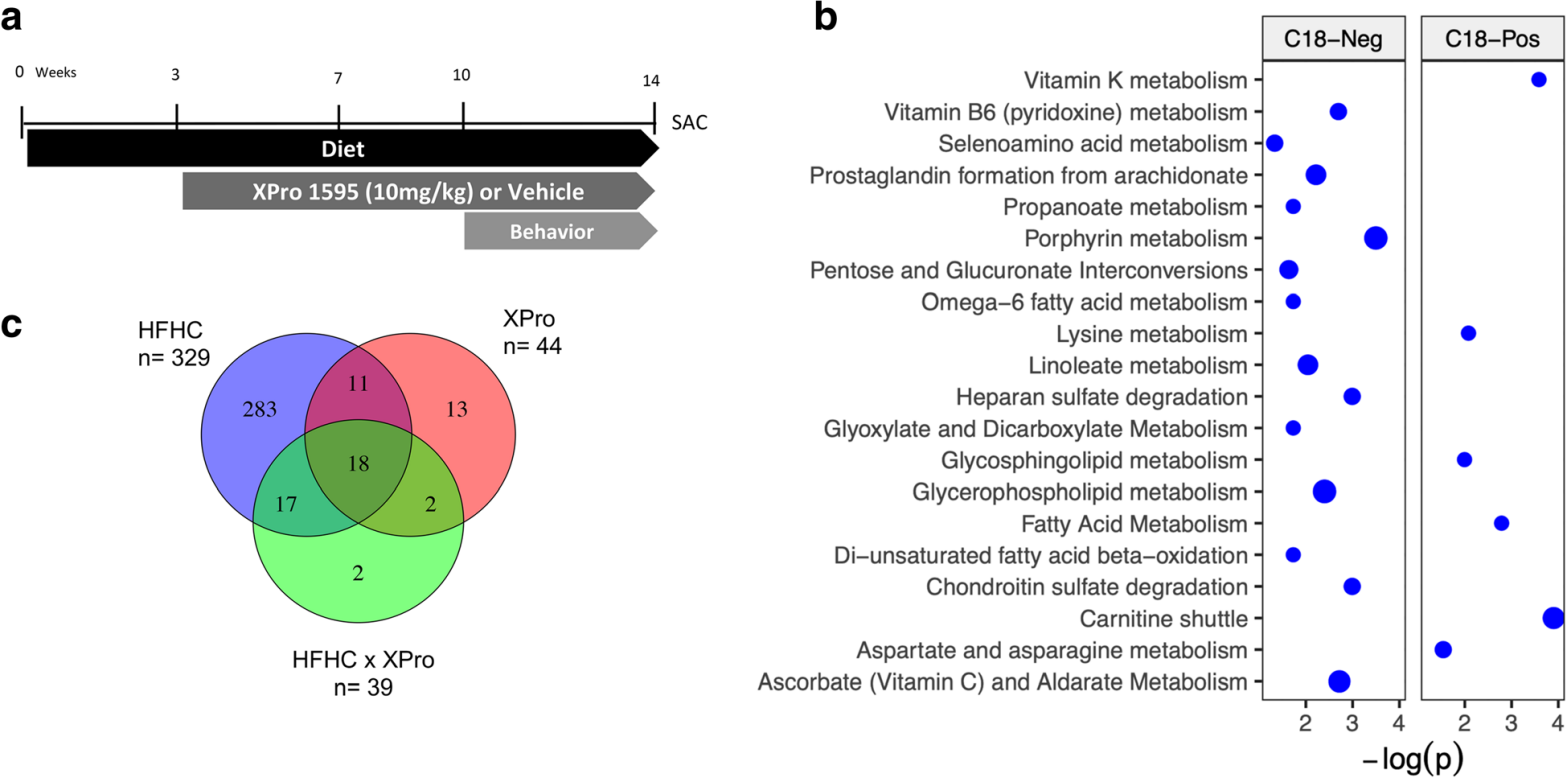
Plasma metabolic profile associated with HFHC diet consumption and solTNF neutralization with XPro1595. **a** Graphic representation of the study timeline. Diet—chow (4% kcal + water) or HFHC (high fat 42% kcal fat + 30% fructose w/v). Injections—subcutaneous injections every third day. Behavior—sociability, open field, and marble burying. At 14 weeks, mice were euthanized, and tissues were harvested (*n* = 12–13/group). **b** Mummichog pathway enrichment of HFHC differentially expressed *m/z* features included pathways related to amino acid metabolism, fatty acid and lipid pathways, oxidative stress, and pentose/glucuronate and proteoglycan metabolism. **c** Untargeted high-resolution metabolomics was used to identify plasma metabolic changes associated with HFHC diet, solTNF neutralization, and the interaction between the two. The majority of *m/z* features identified using two-way ANOVA with post hoc analysis were differentially expressed in association with the HFHC diet. Associations with solTNF neutralization and the interaction between diet and solTNF were also detected. Venn diagram quantities represent the number of mass spectral features with FDR < 5% prior to the metabolite annotation, *n* = 7–13 animals/group

After 3 weeks of diet treatment, animals received subcutaneous injections of the selective inhibitor of soluble TNF XPro (10 mg/kg in the saline vehicle) or saline every third day for 11 weeks. Mice were randomly assigned to one of the following treatment groups (*n* = 12–13 per group): control diet saline (CD Saline), control diet XPro (CD XPro), high-fat high-carbohydrate diet/saline (HFHC Saline), and high-fat high-carbohydrate diet XPro (HFHC XPro) (Fig. 1a). Mice were brought up to the lab 4 h prior to the endpoint at which point food was removed from the cage. All animals were sacrificed in the early morning in the middle of their inactive period approximately 6 h after the food was withdrawn from their cages. Following euthanasia liver, retroperitoneal and gonadal adipose tissues were collected and weighed for assessment of lipid deposition. The small intestine and colon lengths were measured, as the shortness of the intestines is associated with gut inflammation in mice [23]. Tissue samples were frozen in liquid nitrogen and stored at − 80 °C. All experiments were performed by blind experimenters.

### Multiplexed immunoassays and metabolic measurements

The trunk blood was collected in EDTA and centrifuged at 2000*g* for 15 min at 4 °C. The plasma was collected and stored at − 80 °C until measurements for LCN2 (Lipocalin-2/NGAL Quantikine ELISA Kit, R&D Systems), IL-1, IL-6 (Mouse Proinflammatory 7-Plex Ultra-Sensitive Kit, Meso Scale Discovery immunoassays), triglycerides ELISA (Charles River Lab), total cholesterol (Cholesterol Quantitation Kit, Sigma-Aldrich), and insulin and leptin (Mouse Metabolic Kit (Multi-spot Assay System, Meso Scale Discovery). Plates were processed in a SECTOR® Imager 6000 plate reader (Meso Scale Diagnostics, LLC). Data acquired using the Discovery Workbench software (v4.0; Meso Scale Diagnostics, LLC).

### Metabolomics

To gain further insight into the resultant metabolic and immune alterations present in the adipose tissue and in the gut-liver axis in diet-induced insulin impairment, we next assessed the expression of metabolites in plasma and liver tissue. Plasma and liver tissue samples were analyzed for untargeted metabolomics using established methods [24]. For the analysis of plasma, 50 μL was treated with 100 μL of acetonitrile to precipitate proteins, and triplicate 10-μL aliquots were analyzed by C18 (Accucore, 100 mm × 2.1 mm, 2.6 μm; Thermo Scientific) chromatography with acetonitrile/formic acid gradient interfaced to a Q-Exactive HF high-resolution mass spectrometer (Thermo Scientific) operated in a positive and negative electrospray ionization (ESI) mode. Polar fractions collected from liver samples were analyzed using HILIC chromatography for comparison of tissue metabolites to plasma results. Mass spectral signals were detected using a Thermo Fusion high-resolution mass spectrometer operated at a resolution of 120,000 and scan range 85–1250. Raw data for both plasma and tissue metabolomics analysis was processed using apLCMS with modifications by xMSanalyzer, which provided a feature table of uniquely detected features consisting of mass-to-charge ratio (*m/z*), retention time, and peak intensity. Prior to statistical analysis, replicate injections were averaged, log_2_ transformed, and filtered to remove features with greater than 20% missing values. Following statistical analysis, altered pathways were identified using Mummichog and all *m/z* features meeting the false discovery rate (FDR) threshold.

### Western immunoblotting

Immunoblot analyses were performed as previously described [25]. Flash-frozen samples were stored at − 80 °C until processing. Protein was isolated from the hypothalamus, PFC, and liver samples with RIPA buffer (1% Triton-X 100, 50 mM Tris HCL, 0.1% sodium dodecyl sulfate, 150 mM NaCL, pH 8.0). Intestinal proteins were isolated using TRIzol (Life Technologies #15596-018). RIPA samples were centrifuged at 12,000 rpm for 20 min at 4 °C. The supernatant was transferred to a new tube for bicinchoninic acid protein assay (Pierce Scientific #23225). TRIzol samples were resuspended in 1% SDS. Samples were diluted to 1 μg/μL in 4× sample buffer (BioRad #1610747) and boiled at 90 °C for 5 min). After BCA analysis, the membranes were probed overnight with ZO-1, OCLN, CLDN2, IRS-1, p-IRS^Tyr608^, p-IRβ^Tyr1150/1151^, p-IRS^Ser307^, p-Akt^Ser473^, p-Akt^Thr308^, AKT, or anti-β-actin primary antibodies (Additional file 4: Table S2). The membranes were exposed to species-appropriate horseradish peroxidase (HRP)-conjugated secondary antibody (1:1000). Bands were visualized by chemiluminescence, and protein band optical intensity was measured using densitometric analysis (Image Studio Lite). Values were normalized relative to β-actin levels from the same sample. The densities of the phosphorylated protein bands were measured relative to the targeted total protein levels.

### qPCR assay

RNA was isolated from the colon, small intestine, liver, hypothalamus, and hippocampus as published previously [25]. Samples were homogenized in TRIzol reagent (Life Technologies). RNA was isolated using the RNeasy mini kit (QIAGEN), and reverse transcription of RNA was performed using SABiosciences RT2 First Strand Kit. qPCR was performed using an ABI Prism 7900HT Fast Detection System (Applied Biosystems). Primers were designed using Primer-Blast (www.ncbi.nlm.nih.gov/tools/ primer-blast/). qPCR was performed as published previously [25]. Relative gene expression was measured by validated primers (Additional file 5: Table S3) for Lipocalin-2, tight junction protein 1, Occludin, Claudin-2, tumor necrosis factor, interleukin 1 beta, interleukin 6, Toll-like receptor 2, Toll-like receptor 4, suppressor of cytokine signaling 3, peroxisome proliferator-activated receptor alpha, and sterol regulatory element-binding protein-1c (Integrated DNA Technologies). Transcript abundance was quantified using the 2^−ΔΔCt^ method.

### RT^2^ PCR array

Because sustained central inflammation is considered a risk factor for neuroinflammation and neurodegenerative conditions such as AD [19], a RT^2^ PCR profiler was used to investigate the effects of HFHC consumption and solTNF signaling in the hippocampal gene expression. The hippocampus is a brain structure associated with a cognition that is sensitive to high-fat diet-induced insulin resistance [26]. Hippocampal tissue was processed using Qiagen RNeasy mini kit as described previously [25]. After mixing the cDNA template with the appropriate PCR master mix, reverse transcription of RNA was carried out using SABiosciences RT2 First Strand Kit and qPCR was performed using an ABI Prism 7900HT Fast Detection System (Applied Biosystems). Reactions were performed in the 384-well format mouse inflammatory response and Receptors RT2 Profiler PCR Array (PAMM-077Z, SABiosciences). Data analysis was based on the ΔΔCT method with normalization of the raw data to either housekeeping genes.

### Sociability test

A three-chambered sociability apparatus (acrylic, 60 × 40 × 22 cm) (UGO BASILE s.r.l.) was used to assess social interaction as previously described [22]. The total duration of active contact made by the tested mouse was recorded in an area of 3 cm around the mesh empty cup or a cup containing a novel mouse. EthoVision XT (Noldus) was used for behavior analysis. Preference for novel mouse was calculated as [(time spent exploring novel mouse)/(total time spent exploring empty cup and novel mouse)] × 100. Preference for novel object was calculated as [(time spent exploring empty cup)/(total time spent exploring empty cup and novel mouse)] × 100.

### Marble burying test

A marble burying test was conducted as previously described [25] to determine whether HFHC diet and solTNF signaling impact anxiety-like behavior. Mice were placed in a plastic tub (50.5 × 39.4 × 19.7 cm) containing 5 in. of lightly pressed bedding. On top of the bedding, 20 marbles of uniform size and color were placed in 5 rows of 4 marbles each. Mice were placed in the containers and allowed to roam freely for 30 min. At the end of testing, the mice were placed back in home cages, and the number of marbles buried at least two thirds of their height was considered buried.

### Open field test

In the open field test, a mouse that spends less time in or hesitates to re-enter the open center of the testing chamber is considered to be exhibiting anxiety-like behavior [27]. During the light phase of the light/dark cycle, mice were placed into the open field (45 cm × 45 cm square box) and allowed to move freely for 20 min. Distance, velocity, center, and border statistics were measured using the Noldus/Ethovision software. Center was defined as the central 22.5 cm × 22.5 cm.

Stressful behavioral tests such as fear conditioning and Morris water maze were avoided in this study design because our previous study demonstrates the effects of stress on diet-induced insulin resistance [25].

### Histology

Next, because hepatic lipid accumulation leads to insulin impairment and insulin insensitivity, we assessed how HFHC diet consumption and solTNF signaling impact ectopic lipid deposition in liver tissue [28] Liver tissue from the left lobe was fixed in 4% paraformaldehyde/PBS and cryoprotected in 30% sucrose solution. Tissue was frozen in OCT, sectioned (10 μm), and stained with Oil Red O (150678, Abcam) according to the manufacturer’s instructions. Images were obtained using a Nikon Eclipse 90i microscope with a DS-Fi1 (Nikon) camera and Nikon NIS-Elements AR 3.10 software, magnification × 40.

### Statistical analyses

Data are represented as the mean ± standard error of the mean (SEM). For statistical comparisons between the groups, two-way ANOVA followed by Turkey’s post hoc test was used where applicable. Metabolomic features were tested for differential expression using a Benjamini-Hochberg false discovery rate threshold of 5%; for all other analyses, 0.05 *P* value threshold was considered statistically significant. The association between variables was analyzed using the Pearson’s correlation coefficient (*r*). Analyses were performed using GraphPad Prism 6 except where otherwise specified. HRM profiling data were analyzed using R [29]. Metabolites associated with HFHC diet, XPro treatment, and HFHC diet:XPro interations were evaluated using linear models for microarray data (LIMMA) based on two-way ANOVA analysis [30], as implemented in xmsPANDA [31]. Using this approach, metabolites associated with HFHC were identified by comparison of all HFHC-fed mice and control diet mice, irrespective of XPro treatment; metabolites associated with XPro treatment were identified by comparison of all treated mice to saline-fed control; the influence of XPro on diet-associated metabolic changes was evaluated through post hoc tests for each metabolite. Pearson’s correlation coefficient (*r*) was used to analyze the associations between variables. PCR array data were analyzed using the RT2 Profiler TM PCR Array Data Analysis software on the SABiosciences website http://www.sabiosciences.com/pcrarraydataanalysis.php and are expressed as a fold regulation change.

## Results

### solTNF neutralization decreases insulinemia in diet-induced metabolic inflammation

As expected, HFHC-fed mice exhibited significant bodyweight gain compared to control diet (CD) groups starting in the third week of diet (Additional file 1: Figure S1A). Fourteen weeks of HFHC diet was associated with reduced caloric efficiency (Additional file 1: Figure S1B) (*P* < 0.0001), increased bodyweight gain (*P* < 0.0001) (Additional file 1: Figure S1C), and weight of retroperitoneal (*P* < 0.0001) and gonadal fat pads (*P* < 0.0001) (Additional file 1: Figure S1D, E). HFHC diet decreased mesenteric tissue weight (*P* < 0.0001) in both HFHC diet/saline and HFHC diet/XPro groups (Additional file 3: Figure S2F). HFHC diet promoted metabolic dysregulation (Table 1) evidenced by increased plasma cholesterol (*P* < 0.0001), leptin (*P* < 0.0001), and insulin levels (*P* = 0.0005). solTNF blocking promoted a decrease in circulating insulin in animals given HFHC diet (*P* = 0.007). In HFHC mice, increased plasma levels of the acute phase protein LCN2 (*P* < 0.0001) was demonstrated as well as increase in classical pro-inflammatory cytokines IL-6 (*P* = 0.0001) (Table 1) and TNF (*P* = 0.0072) (Additional file 3: Figure S2G). solTNF neutralization decreased LCN2 levels in the HFHC diet group (*P* = 0.0397) and reduced IL-6 in the HFHC-fed mice to levels statistically indistinguishable from CD-fed mice. HFHC diet decreased plasma triglycerides in both HFHC-fed groups (*P* = 0.0057) (Table 1). It is not possible to measure the levels of endogenous solTNF after administration of XPro because the anti-mouse TNF immunoassay captures mouse solTNF homotrimers and heterotrimers alike. 
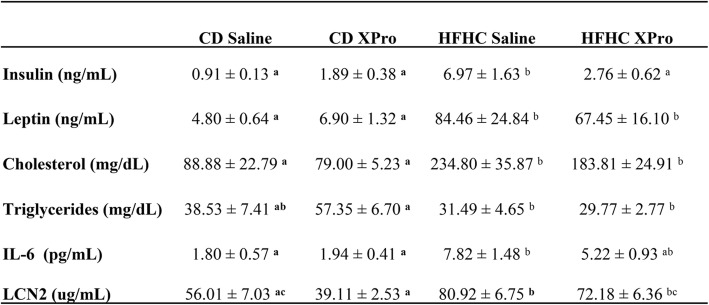

**Table 1:** Soluble TNF neutralization reverts increased insulin plasma levels in presence of diet-induced metabolic inflammation

	CD Saline	CD XPro	HFHC Saline	HFHC XPro
Insulin (ng/mL)	0.91 ± 0.13 ^**a**^	1.89 ± 0.38 ^**a**^	6.97 ± 1.63 ^b^	2.76 ± 0.62 ^a^
Leptin (ng/mL)	4.80 ± 0.64 ^**a**^	6.90 ± 1.32 ^**a**^	84.46 ± 24.84 ^b^	67.45 ± 16.10 ^b^
Cholesterol (mg/dL)	88.88 ± 22.79 ^**a**^	79.00 ± 5.23 ^**a**^	234.80 ± 35.87 ^b^	183.81 ± 24.91 ^b^
Triglycerides (mg/dL)	38.53 ± 7.41 ^**ab**^	57.35 ± 6.70 ^**a**^	31.49 ± 4.65 ^b^	29.77 ± 2.77 ^b^
IL-6 (pg/mL)	1.80 ± 0.57 ^**a**^	1.94 ± 0.41 ^**a**^	7.82 ± 1.48 ^b^	5.22 ± 0.93 ^ab^
Lcn2 (ug/mL)	56.01 ± 7.03 ^**ac**^	39.11 ± 2.53 ^**a**^	80.92 ± 6.75 ^**b**^	72.18 ± 6.36 ^bc^

*Values are presented as an average with standard deviation. Data were analyzed by two-way ANOVA followed by Tukey’s multiple comparisons in GraphPad Prism 6. Letters indicate post hoc analysis. Means with different letters are significantly different from each other, *P* < 0.05. ELISA measurement of fasting plasma collected at endpoint. Insulin (*n* = 10, diet effect *P* = 0.0005, XPro effect *P* = 0.007), leptin (*n* = 10, diet effect *P* < 0.0001), cholesterol (*n* = 5–6, diet effect *P* < 0.0001), triglycerides (*n* = 5–7, *P* = 0.0057), IL-6 (*n* = 10, diet effect *P* = 0.0001), and LCN2 (*n* = 10, diet effect *P* < 0.0001, XPro effect *P* = 0.0397)

### HFHC diet and solTNF signaling impact neuroactive metabolites processing

An untargeted plasma and liver metabolomics was used to investigate the effect of HFHC diet on metabolic profiles and the extent to which solTNF neutralization reversed any of those alterations. The results for all significant metabolites and tissues are provided in Additional files 2, 4, and 5: Tables S4, S5, and S6. HFHC diet promoted significant changes in the plasma metabolic profile, with 329 *m/z* features differentially expressed between the control and HFHC diet (Fig. 1b). Metabolic pathway enrichment identified 20 altered pathways associated with HFHC consistent with proteoglycans, amino acids, lipids and inflammatory lipid pathways, β-oxidation, oxidative stress, and pentose/glucuronate metabolism (Fig. 1c). Comparison of anti-solTNF and saline-treated mice detected 44 *m/z* features associated with solTNF neutralization. Pathway enrichment identified 3 altered pathways, including prostaglandin formation from arachidonate, glycerophospholipid metabolism, and C21-steroid hormone biosynthesis and metabolism. Thirty-nine *m/z* features showed an interaction between HFHC diet and solTNF neutralization. Annotated metabolites included oxidized fatty acids, sterols, bilirubin, and chondroitin 4-sulfate, a metabolite related to proteoglycan synthesis. No specific pathways were associated with the interactions between HFHC diet and solTNF neutralization.

**Fig. 2:**
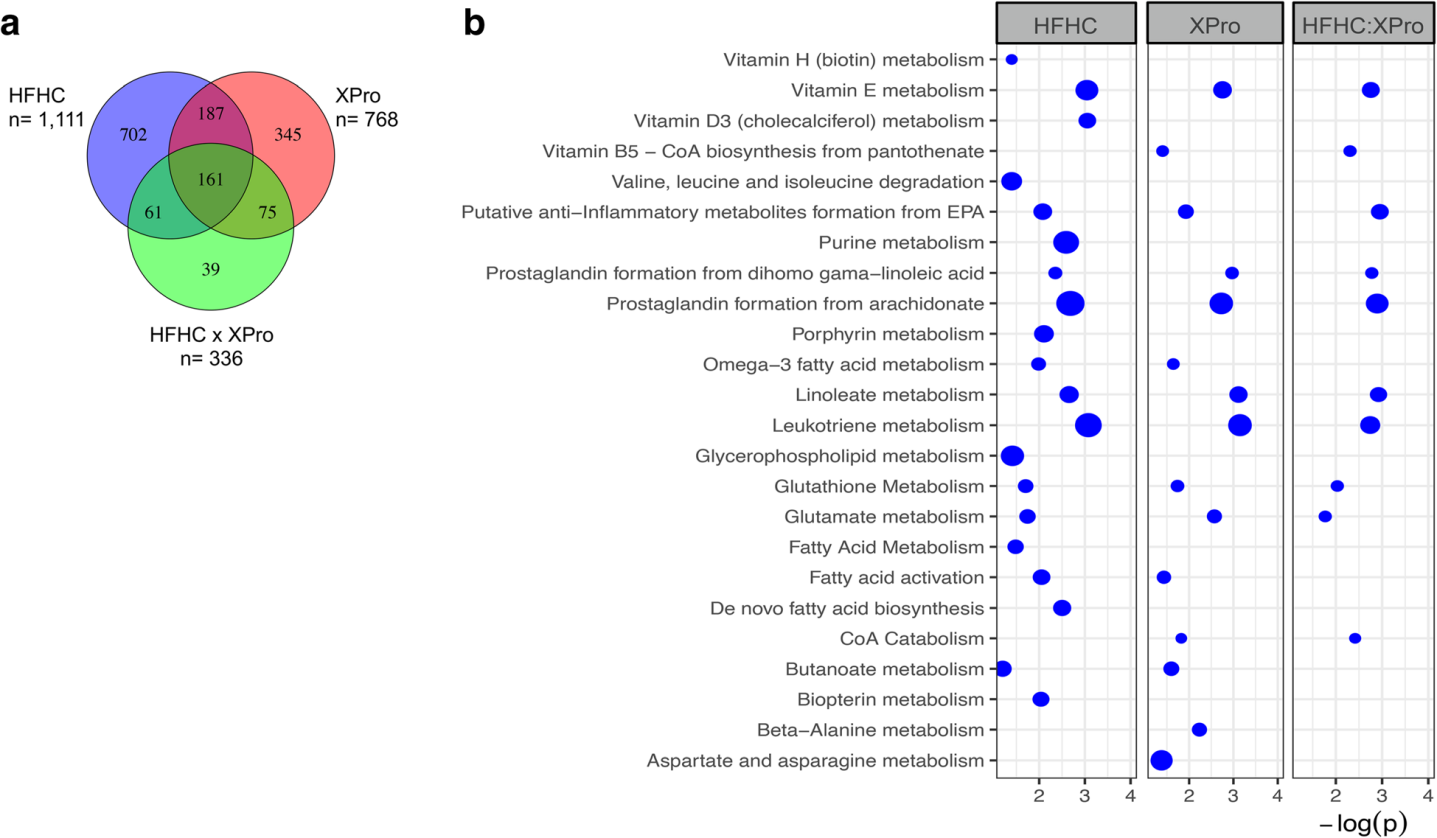
Liver metabolic profile associated with HFHC diet consumption and solTNF neutralization with XPro1595. **a** Untargeted high-resolution metabolomics of hepatic tissues showed a significant metabolic response to diet and solTNF treatment. HFHC showed the greatest number of metabolic changes, with 1111 *m/z* features differentially expressed in association with diet. Targeting solTNF promoted marked liver metabolic alterations and resulted in differential expression of 768 *m/z* features. A large number of these showed interaction with diet or were also associated with hepatic changes following HFHC diet consumption. Venn diagram quantities represent the number of mass spectral features with FDR < 5% prior to metabolite annotation. **b** Mummichog pathway enrichment of *m/z* features associated with HFHC showed changes in inflammatory, lipid, oxidative stress, cofactor, branched-chain amino acid, biopterin, and purine pathways. solTNF treatment showed association with a number of these pathways, including inflammatory, oxidative stress, and fatty acid pathways. Interaction between solTNF and HFHC suggests solTNF neutralization could mediate diet-induced changes in inflammation and oxidative stress in hepatic tissues. Analysis using two-way ANOVA with post hoc analysis at false discovery rate (FDR) threshold ≤ 5%, *n* = 12 animals/group

Two-way ANOVA of liver tissues identified unique metabolic phenotypes of HFHC, solTNF neutralization and the interaction between the two, 1111 *m/z* features that were differentially expressed with HFHC diet, and 336 *m/z* features associated with the interaction between HFHC diet and solTNF neutralization (Fig. 2a). HFHC was also associated with disruptions of hepatic metabolites associated with insulin impairment such as biopterin, branched-chain amino acid, and purine metabolism (Fig. 2b). Neuroactive metabolites and intestinal bacterial products such as butanoate, propanoate, and glutamate were associated with HFHC impact on liver tissue. Interaction between solTNF neutralization and HFHC pathways suggests that solTNF neutralization could influence some of the metabolic effects of HFHC diet in the liver (Fig. 2b). solTNF blocking promoted marked liver metabolic alterations in the CD group in the absence of chronic inflammation. These findings highlight the importance of the regulatory effects of solTNF signaling in the hepatic metabolism.

### solTNF inhibition decreases hepatic LCN2 in the presence of diet-induced insulin impairment and liver inflammation

**Fig. 3:**
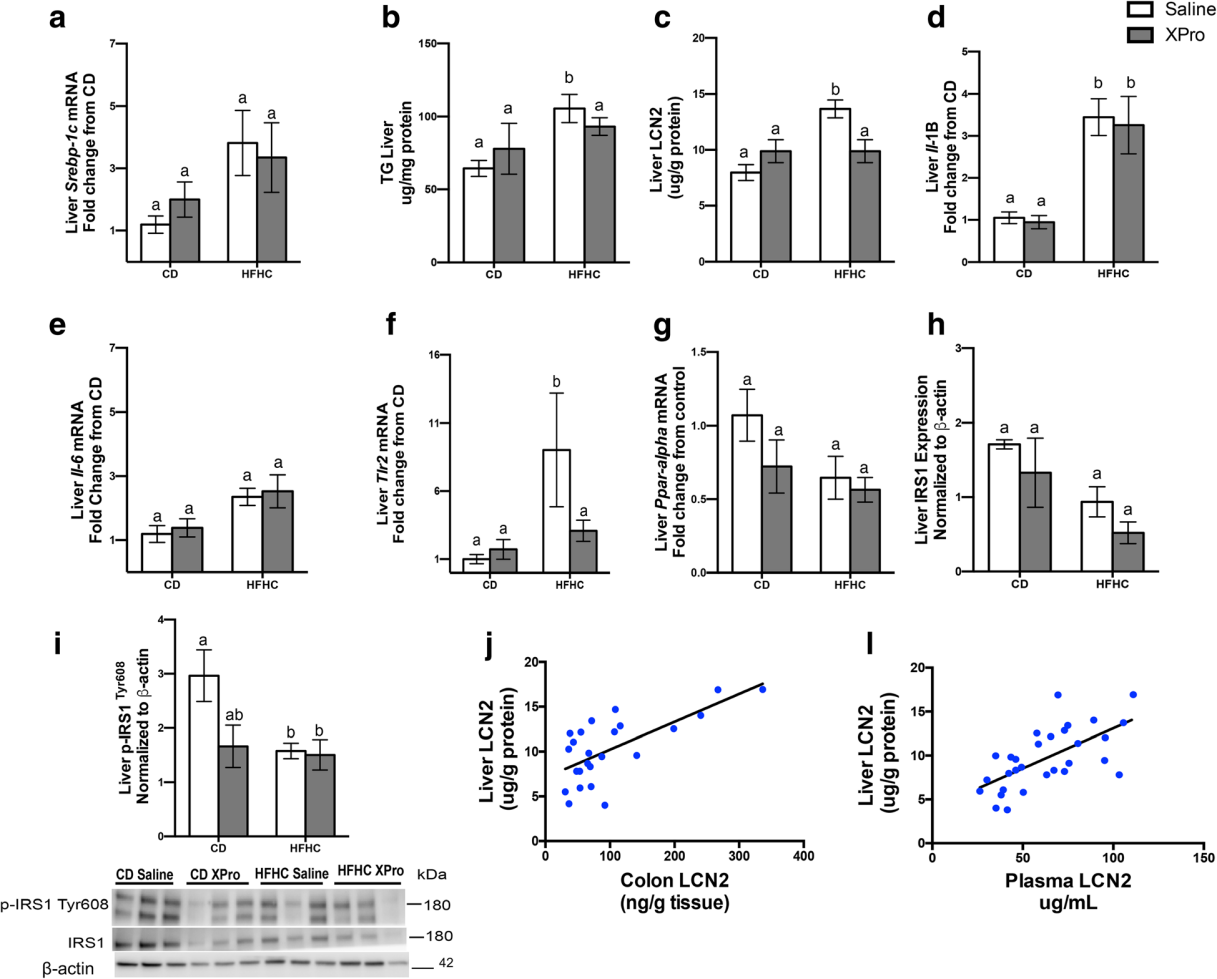
solTNF neutralization decreases hepatic LCN2 in diet-induced liver inflammation. **a** HFHC diet impact on *Srebp-1c* mRNA expression (*n* = 6, diet effect *P* = 0.0263). **b** Increased hepatic triglyceride accumulation was observed in the HFHC groups (*n* = 8, diet effect *P* = 0.0102). **c** solTNF neutralization decreases elevated hepatic LCN2 associated with HFHC diet (*n* = 9–11, diet effect *P* = 0.0034, interaction *P* = 0.0034). **d**-**g** HFHC diet impact on *Il-1* (*n* = 6, diet effect *P* < 0.0001), *Il-6* (*n* = 6, diet effect *P* = 0.0036), *Tlr2* (*n* = 5–6, diet effect *P* = 0.0221), and *Ppar-α* (*n* = 5–6, diet effect *P* = 0.0348) liver mRNA expression in the HFHC groups. **h**, **i** Immunoblot analysis demonstrates decreased IRS1 expression (*n* = 6, diet effect *P* = 0.0075) and phosphorylated of IRS1 (TYR608) (*n* = 6, diet effect *P* = 0.0372) after 14 weeks of HFHC diet treatment. **j**-**l** Scatter plots indicate significant Pearson correlation analysis (*r* value) between hepatic and colonic LCN2 levels (*r*^2^ = 0.4650, *P* < 0.0002) and hepatic and plasma LCN2 (*r*^2^ = 0.4168, *P* < 0.0001). Tissues were analyzed by qPCR using primers directed against murine Il-1, Il-6, Tlr2, and Ppar-alpha. RNA expression. For each animal, the Ct values were normalized to the Ct values for Gapdh and Ppia. The relative expression level of the target gene (fold change) was expressed as 2^−ΔΔCt^, when compared with the mean DCt (threshold cycle) of the control group. Immunoblots are representative of two independent experiments. Band intensity was calculated using Image Studio Lite, and values were normalized to the intensity of β-actin. Blot images were cropped for comparison. Data were analyzed by two-way ANOVA followed by Tukey’s multiple comparisons in GraphPad Prism 6. Data in bar graphs are represented as the mean ± SEM. Lowercase letters indicate post hoc analysis. Values with different lowercase letters are significantly different from each other. Means with different lowercase letters are significantly different from each other, *P* < 0.05

LCN2 is a downstream TNF inflammatory molecule associated with hepatic steatosis and insulin insensitivity [32]. To gain further insight into the resultant immune alterations present in the gut-liver axis, we next assessed the hepatic LCN2 levels and the expression of inflammatory factors in the liver tissue. Differences in macroscopic gross liver appearance (Additional file 3: Figure S2 A-D) and liver weight (Additional file 3: Figure S2 E) (*P* = 0.0006) suggested hepatic lipid accumulation associated with HFHC consumption. Specifically, the Oil Red O staining of the liver tissue sections revealed intense lipid deposition in the HFHC groups. This lipid deposition was partially corrected by XPro treatment (Additional file 3: Figure S2C-D). The disturbance in the lipid metabolism was confirmed by the impact of the HFHC diet on sterol regulatory element-binding protein-1c (*Srebp-1c*) mRNA expression (*P* = 0.0263) (Fig. 3a). Additionally, increased hepatic triglyceride accumulation (*P* = 0.0102) was observed in the HFHC diet/saline group compared to the CD group (*P* < 0.0006) (Fig. 3b). We next assessed the hepatic LCN2 levels and the expression of inflammatory factors in the liver tissue. HFHC diet mice developed elevated concentrations of hepatic LCN2 (*P* = 0.034), and a significant interaction between diet and solTNF neutralization was found (*P* = 0.0034), with LCN2 levels in the HFHC diet/XPro group indistinguishable from the CD group (Fig. 3c).

The hepatic inflammatory profile after HFHC diet treatment was confirmed by an increase in hepatic *Il-1b* (*P* < 0.0001) and *Il-6* (*P* = 0.0036) mRNA expression (Fig. 3d, e). Diet treatment raised *Tlr2* (*P* = 0.022) and decreased *Ppara* (*P* = 0.034) mRNA expression compared to the CD groups (Fig. 3f, g). XPro decreased *Tlr2* expression in HFHC diet-treated mice to levels statistically indistinguishable from the CD group. XPro reduced *Ppara* levels in the CD group. There was no significant difference in hepatic *Tlr4* between the experimental groups (Additional file 3: Figure S2F). The assessment of insulin signaling in isolated liver tissue revealed decreased IRS1 phosphorylation at tyrosine 608 (TYR 608) in the HFHC diet/saline compared to the CD group (*P* = 0.0372); a diet effect decreased the expression of IRS1 (*P* = 0.0075) in the liver (Fig. 3h, i). A positive correlation between hepatic and colonic LCN2 (*r*^2^ = 0.4650, *P* < 0.0002) and hepatic and plasma LCN2 levels was observed LCN2 (*r*^2^ = 0.4168, *P* < 0.0001) (Fig. 3j, l).

### solTNF inhibition decreases colonic LCN2 and tight junction protein alterations associated with HFHC diet

**Fig. 4:**
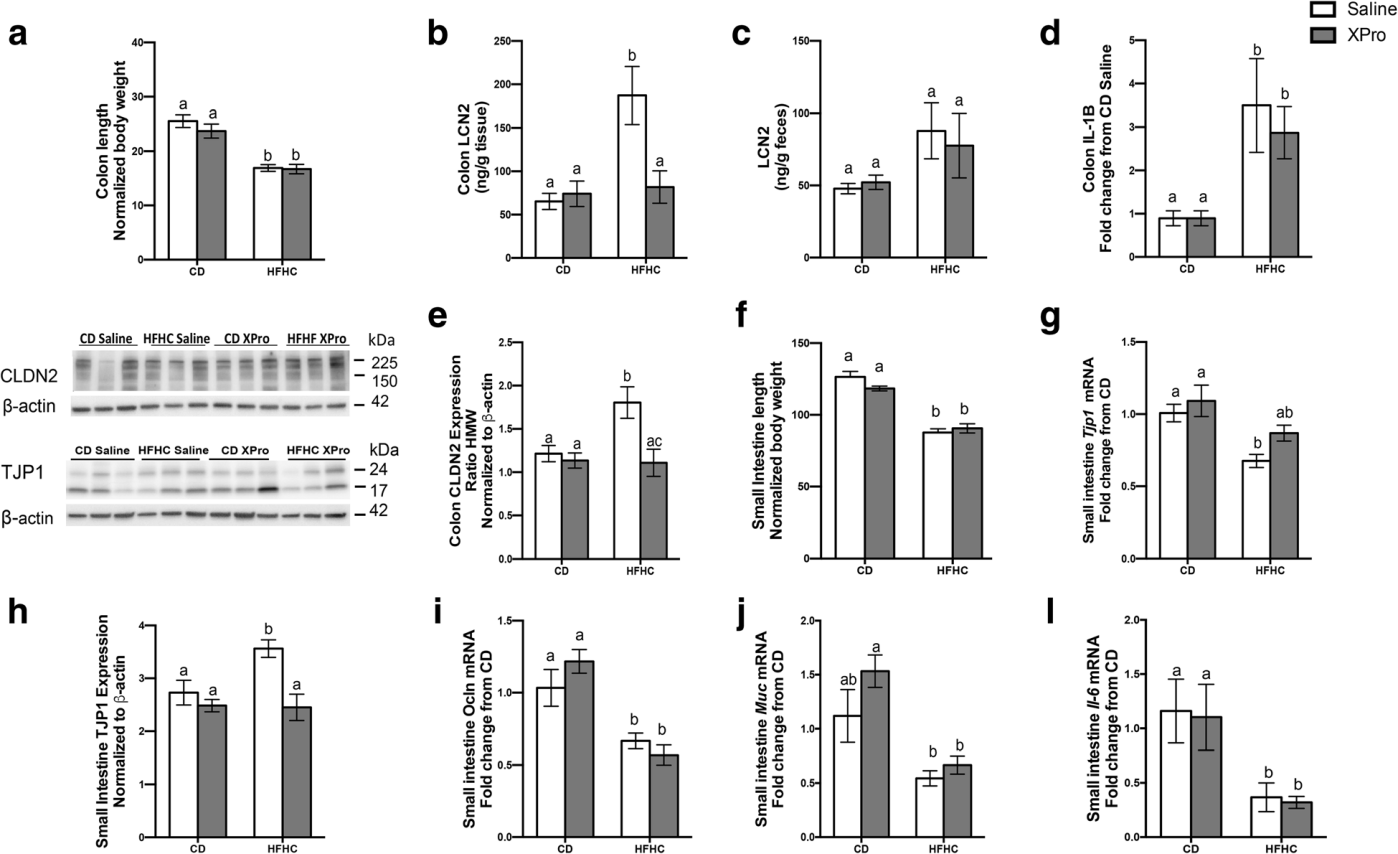
solTNF neutralization with XPro1595 reduces colonic LCN2 and reverses tight junction protein alterations associated with HFHC diet consumption. **a**-**c** HFHC diet promoted and decrease in colon length (*n* = 12–13, diet effect *P* < 0.0001) and an inflammatory colonic profile demonstrated by elevated LCN2 in colon (*n* = 6–9, diet effect *P* = 0.0091) and feces (*n* = 8–10, diet effect *P* = 0.0252). **d** Colonic *Il-1b* mRNA expression (*n* = 6, diet effect *P* < 0.0001). XPro 1595 reverses colonic LCN2 (*P* = 0.0460). **e** solTNF neutralization and HFHC diet-associated alterations in proteins involved in barrier function in the colon (CLDN2 *n* = 6, diet effect *P* = 0.0091), solTNF inhibition decreased the ratio of claudin-2 high/low in the HFHC diet/XPro1595 group (CLDN2 *n* = 6, XPro effect *P* = 0.0425). **f** HFHC diet decreases small intestine length (*n* = 12–13, diet effect *P* < 0.0001). **g**-**i** Small intestinal barrier alterations in HFHC mice (Tjp1 mRNA expression, *n* = 6, diet effect *P* = 0.0012; TJP1 protein expression, *n* = 6, diet effect *P* = 0.05; Ocln mRNA, *n* = 6, diet effect *P* < 0.0001). **j**, **l** *Muc (n = 6, diet effect, P < 0.0001) and Il-6* (*n* = 6, diet effect, *P* = 0.0004) mRNA expression in the small intestine. Tissues were analyzed by qPCR; for each animal, the Ct values were normalized to the Ct values for Gapdh and Ppia. The relative expression level of the target gene ratio of high molecular weight to low molecular weight forms of CLDN2 and TJP1 protein expression was assessed by immunoblot. Immunoblots are representative of two independent experiments. Band intensity was calculated using Image Studio Lite, and values were normalized to intensity of β-actin. Blot images were cropped for comparison. Data were analyzed by two-way ANOVA followed by Tukey’s multiple comparisons in GraphPad Prism 6. Bar height indicates mean of samples; error bars indicate standard error of the mean (SEM). Lowercase letters indicate post hoc analysis. Means with different lowercase letters are significantly different from each other, *P* < 0.05

Having observed decrease in the colon (*P* < 0.0001) (Fig. 4a) and small intestine lengths (*P* < 0.0001) (Fig. 4f) associated with HFHC diet, we next addressed the ability of solTNF neutralization to reverse DIO-related intestinal changes. Animals exposed to HFHC diet developed an inflammatory colonic profile demonstrated by elevated colonic (*P* = 0.0091) and fecal (*P* = 0.0252) LCN2 and *Il-1β* mRNA expression (*P* < 0.0001) (Fig. 4b-d). There was a detectable XPro effect of decreasing colonic LCN2 in the HFHC diet group (*P* = 0.0460) (Fig. 4b). In addition to these inflammatory changes, the HFHC diet and XPro interaction impacted the high/low ratio of the permeability-promoting Claudin-2 protein in the colon (*P* = 0.0091). solTNF inhibition decreased the ratio of Claudin-2 high/low in the HFHC diet/XPro group (*P* = 0.0425) (Fig. 4e). HFHC diet/saline mice exhibited an increase in *Tjp1* (*Zo-1*) mRNA expression (*P* = 0.0007) and decrease of this tight junction protein levels in the small intestine compared to the CD groups (*P* = 0.05) (Fig. 4g, h). Blocking solTNF signaling attenuated the increase in TJP1 protein in the small intestine (*P* = 0.0027) (Fig. 4h). Additionally, HFHC-fed mice presented decreased *Ocln* (*P <* 0.0001), *Muc* (*P* < 0.0001), and *Il-6* (*P =* 0.0004) mRNA expression in the small intestine (Fig. 4i-l).

### HFHC consumption and solTNF neutralization impact the expression of immunomodulatory genes in the brain

**Fig. 5:**
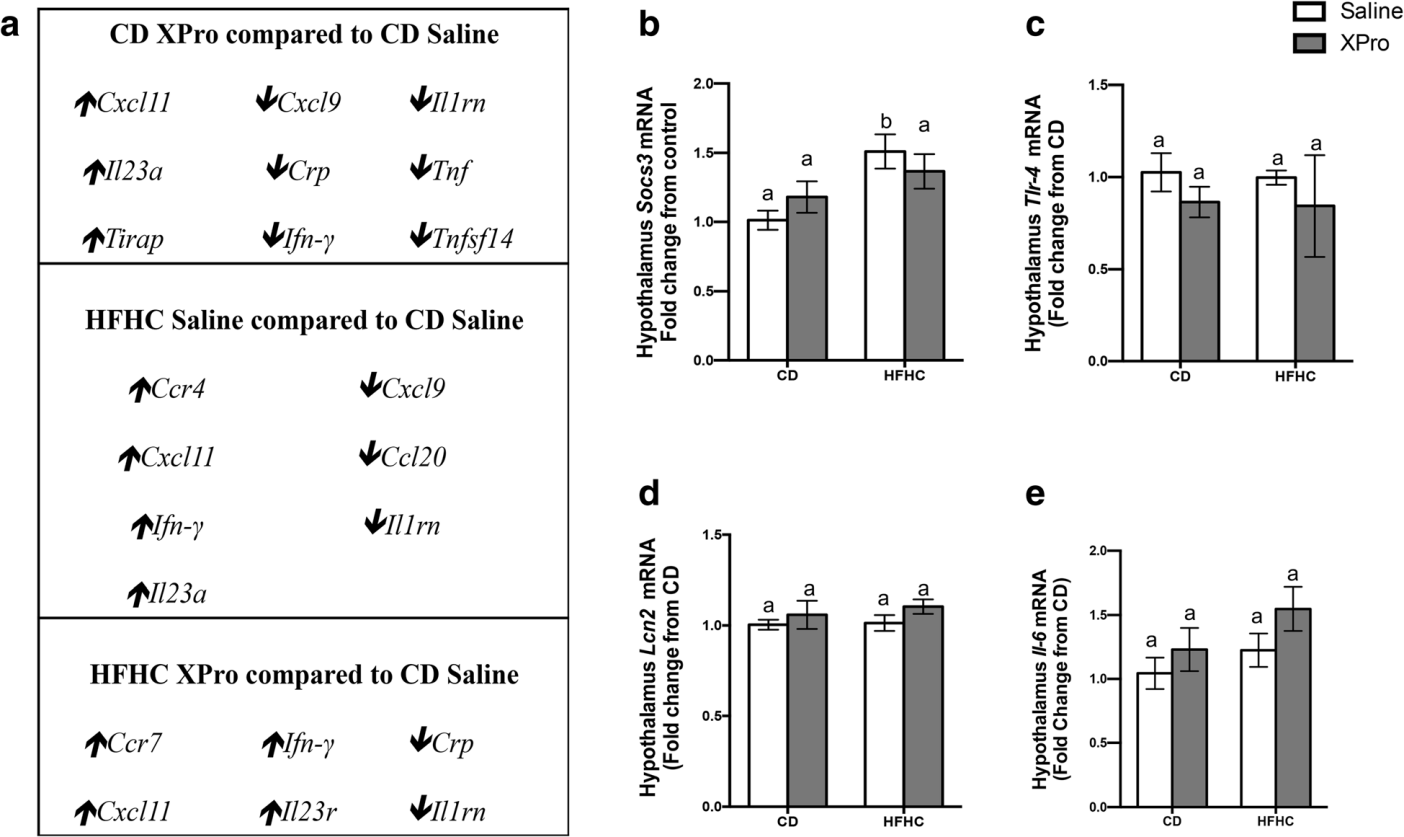
High-fat high-carbohydrate (HFHC) diet and solTNF neutralization with XPro1595 impact brain immunomodulatory genes expression. **a** RT^2^ Profiler PCR Array analysis of the pre-frontal cortex demonstrate immunomodulatory genes significantly up- and downregulated (≥ 3-fold regulations changes) in the pre-frontal cortex in the presence of high-fat high-carbohydrate diet or soluble TNF neutralization. **b**-**e** HFHC diet increases *Socs3 m*RNA expression in the hypothalamus (*n* = 6, diet effect *P* = 0.0018); no significant changes in hypothalamic *Tlr4*, *Lcn2*, and *Il-6* were observed. Tissues analyzed by qPCR had the Ct values normalized to the Ct values for *Gapdh* and *Ppia*. The relative expression level of the target gene (fold change) was expressed as 2^−ΔΔCt^, when compared with the mean DCt (threshold cycle) of the control group. Pre-frontal cortex samples were pooled together from an *n* = 6–7 mice per group. Abbreviations: qPCR, quantitative real-time reverse-transcription polymerase chain reaction; GAPDH, glyceraldehyde 3-phosphate dehydrogenase; *Cxcl11*, chemokine (C-X-C motif) ligand 11; *Cxcl9*, chemokine (C-X-C motif) ligand 9; *Il1rn*, interleukin 1 receptor antagonist; *Il23a*, interleukin 23, alpha subunit p19*; Crp*, C-reactive protein, pentraxin-related; *Tnf*, tumor necrosis factor; *Tirap*, Toll-interleukin 1 receptor (TIR) domain-containing adaptor protein; *Ifn*, interferon; *Tnfsf14*, tumor necrosis factor (ligand) superfamily, member 14; *Ccr4*, chemokine (C-C motif) receptor 4; *Ccl20*, chemokine (C-C motif) ligand 20; *Ccr7*, chemokine (C-C motif) receptor 7. *qPCR* data were analyzed by two-way ANOVA followed by Tukey’s multiple comparisons in GraphPad Prism 6. Bar height indicates mean of samples; error bars indicate standard error of the mean (SEM). Means with different lowercase letters are significantly different from each other, *P* < 0.05

Sustained inflammatory processes in life are associated with cytotoxic consequences and may impact the incidence and acceleration of age-related neuroinflammatory diseases [33]. HFHC diet and solTNF neutralization promoted dysregulation of hippocampal immunomodulatory genes (greater than threefold regulation) (Fig. 5a). Peripheral administration of a brain-permeant solTNF-neutralizing agent in the absence of systemic inflammation promoted upregulation of *Ccx11* (chemokine (C-X-C motif) ligand 11), *Il23a* (interleukin 23, alpha subunit p19), and *Tirap* (Toll-interleukin 1 receptor /TIR domain-containing adaptor protein), and downregulation of *Il1rn* (interleukin 1 receptor antagonist), *Crp* (C-reactive protein), *Tnf*, *Ifn*-*γ* (interferon-gamma), and *Tnfsf14* (tumor necrosis factor ligand superfamily member 14). The gene expression of inflammatory cytokines, cytokines mediating signaling, and chemokines were affected by HFHC consumption and solTNF inhibition (Fig. 5a). HFHC consumption increased the expression of *Socs3* in the hypothalamus. solTNF neutralization decreased hypothalamic *Socs3* RNA expression in HFHC diet-fed mice to levels statistically indistinguishable from CD mice. Increased SOCS3 is associated with deleterious effects of high leptin levels on diet-induced IR [34]. This data is particularly relevant because recent literature indicates that SOCS3, a well-known negative modulator of insulin signaling and immunoregulator, is increased in the brains of individuals with AD, suggesting that SOCS3 may regulate the central insulin signaling pathways that are implicated in neurodegeneration in AD [35]. No significant impact of HFHC diet or solTNF inhibition on hypothalamic *Tlr4*, *Lcn2*, or *Il-6* expression was observed in the experimental groups (Fig. 5c-e).

### Peripheral injections of a solTNF inhibitor revert central insulin signaling impairment and behavioral deficits in DIO

Evidence suggest that systemic and central energy balance is regulated by hypothalamic insulin, which partially occurs by hypothalamic-liver interactions controlling the glucose metabolism [12, 26]. The impact of DIO and solTNF inhibition on central insulin signaling in the hypothalamus and the pre-frontal cortex (PFC) was investigated to evaluate the impact of these metabolic interactions.

**Fig. 6:**
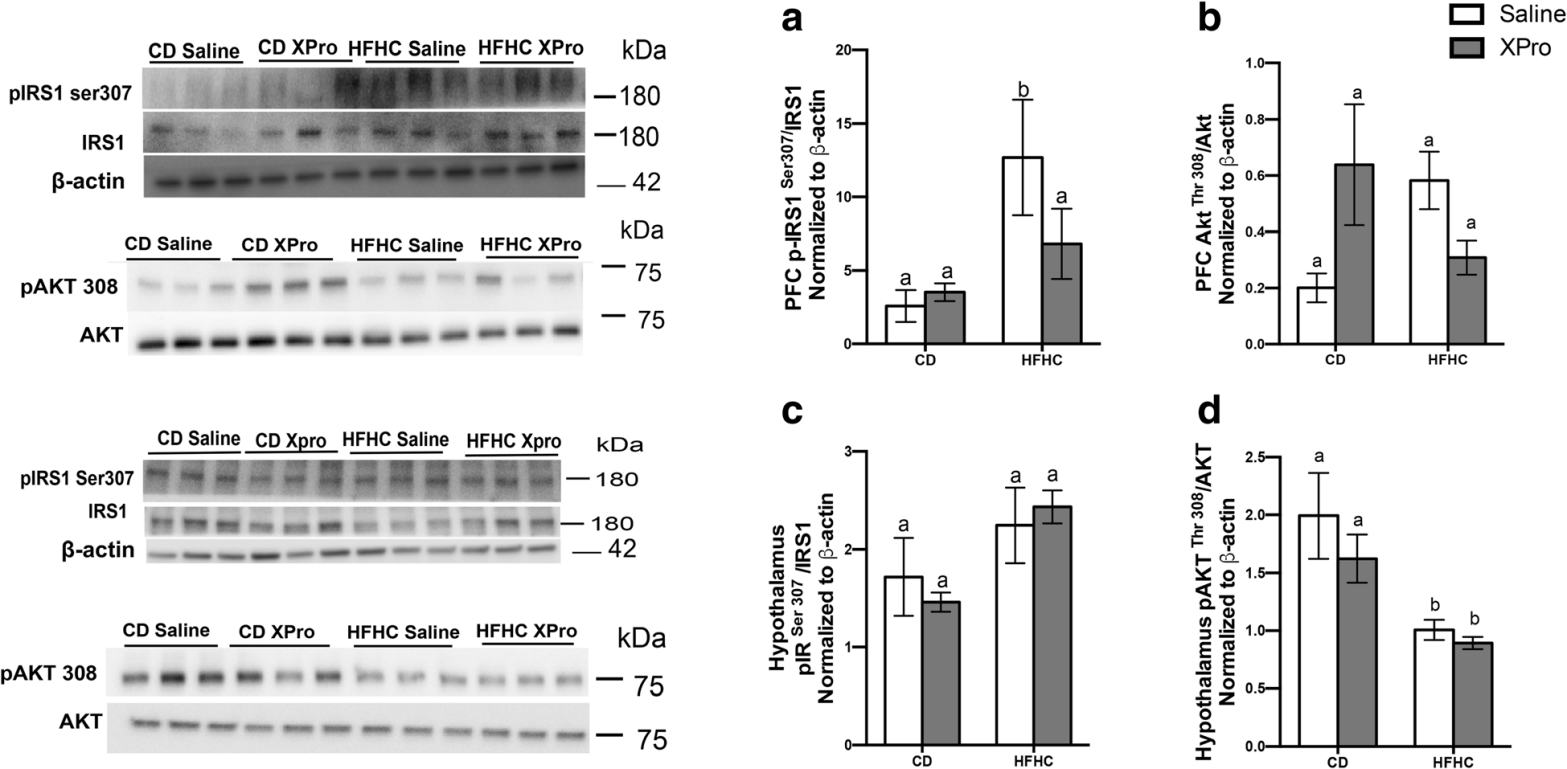
solTNF blockade with XPro1595 reverts diet-induced pre-frontal cortex IRS1/Akt impairment. **a**, **b** HFHC diet increases phosphorylated IRS1 (Ser 307) (*n* = 6, diet effect *P* = 0.0117). HFHC diet XPro 1595 interaction modulated phosphorylated Akt (Thr 308) (*n* = 6, diet effect *P* = 0.0180) in the prefrontal cortex. **c**, **d** Immunoblotting demonstrating the impact of HFHC diet in the hypothalamic phosphorylated IRS1 (Ser 307) (n = 6, diet effect P = 0.0245) and phosphorylated Akt (Thr 308) (*n* = 6, diet effect *P* = 0.0014). Immunoblots are representative of two independent experiments. Band intensity was calculated using Image Studio Lite, and values were normalized to intensity of β-act. Immunoblot images were cropped for comparison. Data were analyzed by two-way ANOVA followed by Tukey’s multiple comparisons in GraphPad Prism 6. Bar height indicates mean of samples; error bars indicate standard error of the mean (SEM). Lowercase letters indicate post hoc analysis. Means with different lowercase letters are significantly different from each other, *P* < 0.05

Increased phosphorylation of p-IRS1 Ser 307 in PFC (*P* = 0.0117) was observed in the HFHC diet/saline group; solTNF inhibition reverted this alteration in HFHC-fed mice to levels statistically indistinguishable from CD-fed mice (Fig. 6a). An XPro and diet interaction modulated p-Akt Thr 308 phosphorylation in PFC (*P* = 0.0180) (Fig. 6b). HFHC diet increased Ser 307 phosphorylation of IRS1 in the hypothalamus (*P* = 0.0245) (Fig. 6c). There was a marked decrease in hypothalamic p-Akt Thr 308 phosphorylation in the HFHC diet-fed groups compared to the CD/saline group (*P* = 0.0014) (Fig. 6d). Indeed, elevated neuronal IRS-1 serine phosphorylation was found in the cerebral cortex of AD subjects and emerges as a major cause of IRS-1 dysfunction in AD [36, 37].

**Fig. 7:**
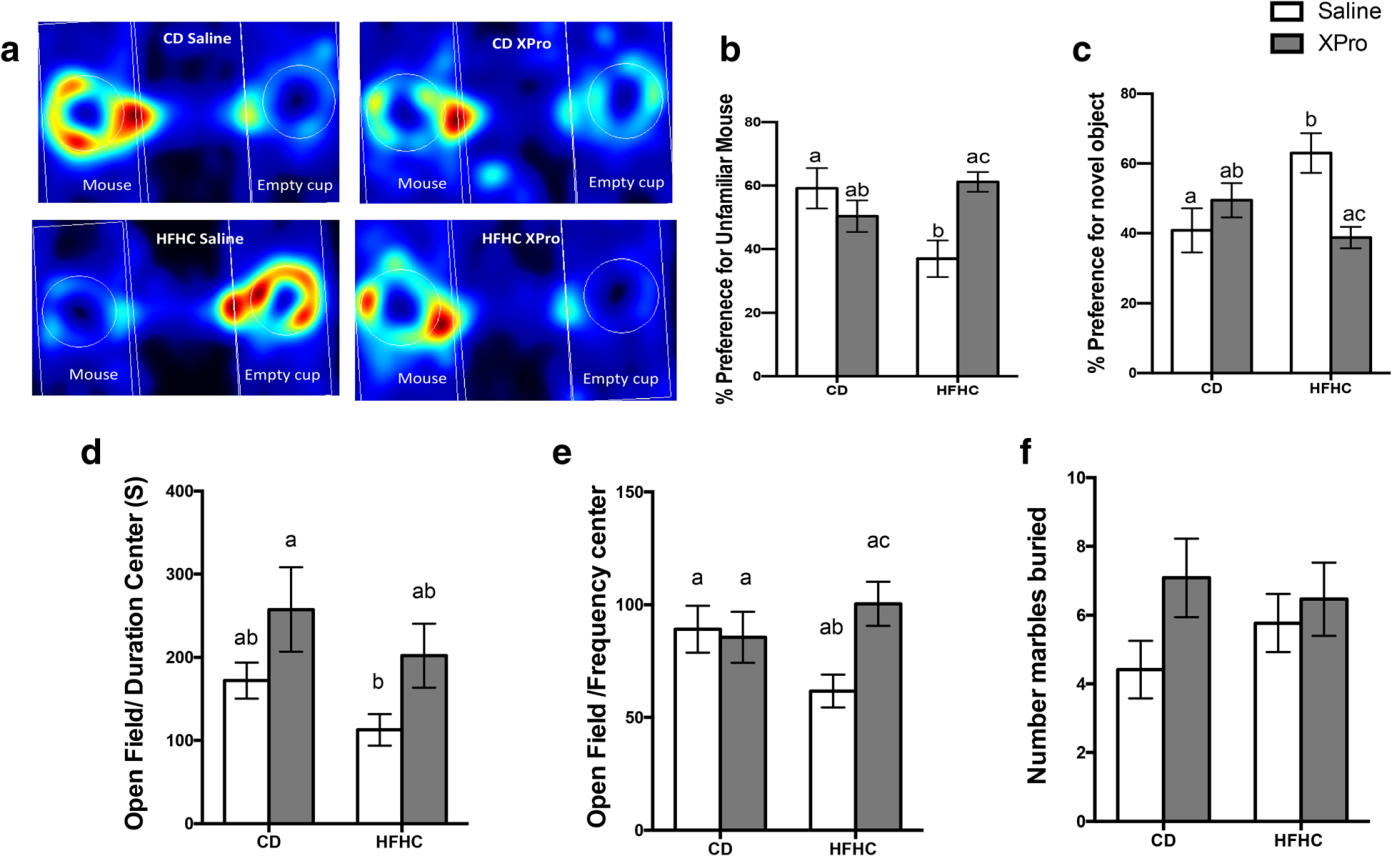
Neutralization of solTNF with XPro1595 prevents the sociability deficits and anxiety-like behavior induced by HFHC diet consumption. **a** Representative heat map demonstrating individual time spent in each chamber of a three-chamber sociability test. **b** HFHC diet decreased the percentage of preference of exploration for an unfamiliar mouse, and solTNF neutralization rescues this behavior alteration (*n* = 11–12, interaction *P* = 0.0027). **c** HFHC-fed mice percentage of exploration of a novel object (*n* = 11–12, interaction *P* = 0.0027). Preference for novel mouse was calculated as [(time spent exploring novel mouse)/(total time spent exploring empty cup and novel mouse)] × 100. Preference for novel object was calculated as [(time spent exploring empty cup)/(total time spent exploring empty cup and novel mouse)] × 100. **d**, **e** Soluble TNF neutralization and its interaction with HFHC diet impacted time (*n* = 12–12, *P* = 0.0152) and frequency (*n* = 12–13, *P* = 0.0349) in the center of an open field as an indication of anxiety-like behavior. Animal behavior activity assessed using EthoVision XT Software. **f** No changes in the number of marbles buried was observed between the groups. Heat map representative of a single experiment. Data were analyzed by two-way ANOVA followed by Tukey’s multiple comparisons in GraphPad Prism 6. Bar height indicates mean of samples; error bars indicate standard error of the mean (SEM). Lowercase letters indicate post hoc analysis. Means with different lowercase letters are significantly different from each other, *P* < 0.05

The three-chamber sociability test allows the evaluation of two different aspects of social behavior: social motivation and social memory and novelty [38]. We previously demonstrated that HFHC consumption promotes social deficits in our animal model of diet-induced insulin resistance [25]. Here, solTNF blocking reverted social deficits in HFHC-fed mice by reducing the percentage of time exploring an empty cup (*P* = 0.0027) and increasing the time spent in social interaction (*P* = 0.0027) (Fig. 7a-c). solTNF neutralization increased time spent in the center of the open field compared in the CD XPro group compared to HFHC saline mice (*P* = 0.0152) (Fig. 7d). The second session of this test is designed to estimate the social memory/novelty (propensity of a mouse to spend time with a previously unfamiliar mouse rather than with a familiar mouse). There was no significant difference between the experimental groups in the social memory test (data not shown). A solTNF blocking and HFHC diet interaction increased the frequency in the center of the open field in the HFHC XPro mice compared to the HFHC saline group (*P* = 0.0349) (Fig. 7e). No significant alterations were observed in the marble burying test between the experimental groups (Fig. 7f).

## Discussion

Elevated levels of TNF have been implicated in IR through multiple mechanisms that include the inhibition of the IRS1 through its serine phosphorylation, inhibition of insulin receptor auto-phosphorylation, and impairment of insulin signaling by the activation of phosphatidylinositol 3-kinase Akt mammalian target of rapamycin pathway [13, 39]. Although the effects of anti-TNF therapies on circulating insulin and glucose levels in patients with severe chronic inflammation are well established [18, 40], past attempts at restoring insulin sensitivity using non-selective TNF antagonists that block both membrane-bound and solTNF failed in the presence of metabolic syndrome or obesity conditions [17, 41]. Importantly, our novel findings indicate that selective solTNF neutralization decreases systemic insulin levels without any significant impact on adiposity or weight gain, an outcome that is opposite to that achieved with several non-selective anti-TNF biologics [11].

Here, we speculate that the impact of selective solTNF neutralization on the plasma insulin levels in DIO may be partially attributable to the XPro effects on hepatic metabolites processing and the hepatic and intestinal decreases in LCN2. IR is attenuated by LCN2 deficiency in animal models, and human studies show a positive association between insulin dysregulation, systemic inflammation, and LCN2 levels [14, 15, 42]. Our findings strongly suggest that solTNF and LCN2 interactions are a potential point of intervention for targeting inflammatory tissue-specific responses in obesity. Hepatic LCN2 is frequently associated with IR and liver alterations in states of positive energy balance [14, 43]. We previously reported that hepatic *Lcn2* overexpression, and increased circulating levels of LCN2 are associated with hepatic steatosis and insulinemia in DIO [25]. The current study obtains evidence that colonic LCN2 is increased by the HFHC diet. These data broadly support the contribution of intestinal inflammation to the state of chronic immune activation present in obesity [44]. Additionally, we are the first to demonstrate that selective solTNF signaling with XPro can regulate the hepatic and intestinal LCN2 levels in the presence of hepatic steatosis and metabolic inflammation in DIO. These findings are consistent with the results of human studies that report a positive impact of anti-TNF therapies in reducing NGAL/LCN2 levels in the presence of intestinal alterations [45, 46].

In colonic epithelial cells, LCN2 expression is induced by IL-17A, IL-22, and TNF [45]. In addition to the TNF effects on LCN2, IL-1β, which is also upregulated in the intestine and liver in our animal model, has been associated with LCN2 release by induction of the transcription factor nuclear factor kappa-light-chain enhancer of activated B cells (NFkB) [47]. Our results indicate that elevated hepatic LCN2 and IL-1β are closely associated with hepatic insulin impairment, hepatic steatosis, and excessive lipid circulation, consistent with previous reports [43, 48]. The beneficial effect of solTNF neutralization in reducing intestinal inflammation in obesity is particularly important because intestinal immune alterations are a recognized contributor to metabolic syndrome comorbidities such as glucose and insulin impairment and CNS neurodegenerative processes [49, 50]. We previously demonstrated that the same HFHC used in the current study increases *Lc*n2 mRNA expression in the hippocampus in the presence of diet-induced insulin resistance [25]. This previous finding in addition to the present results is relevant because LCN2/NGAL is associated with the pro-inflammatory signals that impact AD [16]. LCN2 and its receptors are found in a different human postmortem of the brain regions and in the plasma of AD patients [51–53]. Notably, this TNF-induced molecule sensitizes neurons to toxic effects of amyloid, promotes phenotypic changes in glia, induces CNS chemokines production, and has been implicated in cognitive deficits [51–53]. Previous studies suggest that LCN2 may exacerbate insulin resistance in the brain of aging and cognitive-declined subjects [14, 54].

Central insulin impairment is associated with inflammation, oxidative stress, protein deposition, and alterations in synaptic plasticity. Several of these processes are dysregulated in neurodegenerative disorders [36, 55, 56]. Interestingly, HFHC diet impacts insulin and AKT signaling in the hypothalamus and PFC in the presence of metabolic inflammation, and peripheral injections of a solTNF inhibitor were effective at reducing the impact of HFHC diet on insulin signaling in the PFC and hypothalamic *Socs*3 mRNA expression of mice. Our results provide new and important insight into the role of solTNF in central-peripheral insulin interactions in the states of metabolic inflammation.

While the underlying mechanisms that promote sporadic neurodegenerative disease pathogenesis remain elusive, mounting evidence point to the associations between the disruption of metabolites processing in insulin impairment and neurodegenerative conditions such as AD [57, 58]. Distinct altered metabolic pathways affected by a high-energy diet in this study have been implicated in insulin impairment and the pathogenesis of T2D and neurodegenerative disorders [59, 60]. Additionally, disturbance of the complex gut-liver interactions can impact brain processes and associated central-peripheral energetic balance [61–63]. In this regard, recent human and animal studies revealed an association between purine dysregulation and brain inflammatory alterations and AD [57, 64]. Purines act as extracellular messengers and are involved in energetic pathways, signal transduction, immune regulation, neurotrophism, and neurotransmission. Moreover, neurodegeneration and diabetes progression can be hastened by disturbances in purine signaling. The brain depends partially on metabolites processed in the periphery. For instance, the transport of nucleotides synthesized de novo in the liver from the blood into the neurons and glia is an essential prerequisite for its central metabolic utilization [65]. Cumulative evidence suggest that several environmental factors can affect the intestinal microbiome and the complex regulation of the brain-gut axis (hypothalamic-pituitary-adrenal axis, vagal modulation, and bacteria-derived metabolites) that ultimately may affect neurodegenerative diseases [66]. Among the intestinal bacterial metabolites disturbed here by HFHC diet, propanoate, a short-chain fatty acid, is involved in gluconeogenesis [67] that centrally causes inhibition of energy metabolism in brain GABAergic neurons [68]. Another significant aspect of the impact of the HFHC diet on metabolic pathway alterations relevant to insulin metabolism and neurodegeneration is the alteration in the proteoglycan pathways. Heparan sulfate has been identified as an important key factor in neuroinflammation and in the formation of a neurofibrillary tangle in AD [69, 70]. Additionally, in vitro and in vivo studies report that chondroitin sulfate groups are able to modulate insulin amyloid aggregation and protect the brain against amyloid and advanced glycation product-induced toxicity [71, 72]. Together, these findings demonstrate that HFHC diet promotes the formation of metabolites frequently associated with IR and neurodegeneration [60, 73]. Therefore, our results raise intriguing questions regarding the extent to which DIO impact insulin signaling, energy balance, and immune-metabolic interactions in a solTNF-dependent manner to increase the risk for neurodegeneration.

## Conclusions

Our results suggest that the HFHC diet impacts central insulin signaling and immune-metabolic interactions in a solTNF-dependent manner to increase the risk for neurodegenerative conditions. Our novel findings indicate that selective solTNF neutralization can ameliorate peripheral and central diet-induced insulin impairment and identify lipocalin-2 as a potential target for therapeutic intervention to target inflammation and insulin disturbances in obesogenic environments. The present study provides evidence that solTNF neutralization is associated with a reduction in downstream pro-inflammatory signaling decreasing the risk for the immune and metabolic dysregulation present in obesity.

Collectively, our findings identify solTNF as a potential target for therapeutic intervention in inflammatory states and insulin disturbances in obesogenic environments to lower risk for AD. Studies are underway to confirm and extend published observations that an obesogenic diet can accelerate AD-like phenotypes [74] and to directly test the protective effects of solTNF inhibition in vivo against the development of insulin resistance in the brain. An immunomodulatory approach that selectively targets solTNF is likely to have a positive therapeutic impact on broad metabolic and immune interactions in DIO and, if delivered during mid-life, would positively impact patients with metabolic syndrome and/or obesity who are at risk for developing systemic and neurodegenerative conditions later in life.

## Supplementary information

**Additional file 1: Figure S1.** Impact of HFHC diet consumption on mouse body weight and adiposity. A-F, HFHC diet consumption impacts body-weight gain and caloric efficiency, as well as retroperitoneal, gonadal and mesenteric fat pads. Mice were weighted once a week. Caloric efficiency was obtained by dividing caloric intake (kJ) by changes in body weight. Data were analyzed by two-way ANOVA followed by Tukey’s multiple comparisons in GraphPad Prism 6. Bar height indicates mean of samples from 12-13 mice, error bars indicate standard error of the mean (SEM). Letters indicate post hoc analysis. Means with different letters are significantly different from each other, *P* <0.05.

**Additional file 2: Table S1.** Diet intake*. Values are presented as an average ± s.e.m. Data were analyzed by two-way ANOVA followed by Tukey’s multiple comparisons in GraphPad Prism 6. Letters indicate post hoc analysis. Means with different letters are significantly different from each other, P <0.05, *n*=12-13 mice. Food and drink intake were measured daily for 10 weeks (food was not measured during behavioral assessment).

**Additional file 3: Figure S2.** HFHC diet increases hepatic lipid deposition and liver weight. A-D, Macroscopic appearance and histological sections of representative livers stained for Oil Red O show hepatic lipid accumulation associated with HFHC. E, Increased liver weight (n=12-13) and F, impact on *Tlr4* mRNA expression was observed in HFHC groups (*n*=6). G, HFHC diet increases plasma TNF levels, (*n*=9-10). Oil Red O/Hematoxylin counterstained liver sections, magnification 40X. Images were obtained using a Nikon Eclipse 90i microscope with a DS-Fi1 (Nikon) camera and Nikon NIS-Elements AR 3.10 software (*n*=3 per group). Liver tissue was analyzed by qPCR using primers directed against murine *Tlr4.* For each animal, the Ct values were normalized to the Ct values for *Gapdh* and *Ppia*. The relative expression level of the target gene (fold change) was expressed as 2^-ΔΔCt^, when compared with the mean DCt (threshold cycle) of the CD group. Data were analyzed by two-way ANOVA followed by Tukey’s multiple comparisons and unpaired, two-tailed t-test in GraphPad Prism 6. Data in bar graphs are represented as the mean ± standard error of the mean (s.e.m). Letters indicate post hoc analysis. Means with different letters are significantly different from each other, *P*<0.05. Note that because of the mechanism of action of XPro1595 (i.e. sequestration of solTNF via formation of heterotrimers), it is not possible to accurately measure the effect of XPro1595 on levels of endogenous solTNF after administration of XPro1595 because the anti-mouse TNF immunoassay captures mouse solTNF homotrimers and mouse heterotrimers with XPro1595 alike).

**Additional file 4: Table S2.** Western blot antibodies.

**Additional file 5: Table S3.** Real time PCR oligonucleotide sequences*. Specific gene sequences were obtained from Genbank at the National Center for Biotechnology Information (http://www.ncbi.nlm.nih.gov/). Primer specificity was verified using the Basic Local Alignment Search Tool (http://www.ncbi.nlm.nih.gov/blast/). Tight junction protein 1 (*Tjp1*), Occludin (*Ocln*), Claudin-2 (*Cldn2*), Tumor necrosis factor (*Tn*f); Interleukin 1B and 6 (*Il-1B*) and (*Il-6*); Lipocalin-2 (*Lcn2)*; Toll-like receptor 2 (*Tlr2*), Toll-like receptor 4 (*Tlr4*), Suppressor of cytokine signaling 1 and 3 (Socs1 and Socs3), Peroxisome proliferator-activated receptor gamma (*Ppara*), Sterol regulatory element-binding protein 1c (*Srebp-1c)*, Glyceraldehyde-3-Phosphate Dehydrogenase (*Gapdh*) and cyclophilin E (*Cyclo*).

## Data Availability

Supplementary material is available.

## Abbreviations

AD: Alzheimer’s disease
Akt: Phospho-protein kinase B
CCL20: Chemokine (C-C motif) ligand 20
CCR4: Chemokine (C-C motif) receptor 4
CCR7: Chemokine (C-C motif) receptor 7
CLDN2: Claudin-2
CRP: C-reactive protein
CXCL11: Chemokine (C-X-C motif) ligand 11
CXCl9: Chemokine (C-X-C motif) ligand 9
CYCLO: Cyclophilin E
GAPDH: Glyceraldehyde 3-phosphate dehydrogenase
HFHC: High-fat high-carbohydrate diet
IFN: Interferon
Il-1β: Interleukin 1 beta
Il-6: Interleukin 6
Il1RN: Interleukin 1 receptor antagonist
Il23a: Interleukin 23, alpha subunit p19
IR: Insulin resistance
IRS1: Insulin receptor substrate 1
LCN2: Lipocalin 2
OCLN: Occludin
p-/IRβ^(Tyr1150/1151)^: Insulin receptor beta Tyr1150/1151
p-Akt^SER473^: Phospho-protein kinase B serine 473
p-Akt^THR308^: Hospho-protein kinase B threonine 308
p-IRS1^TYR608^: IRS1 phosphorylation at tyrosine 608
p-IRS1^SER307^: IRS1 phosphorylation at serine 307
PPARα: Peroxisome proliferator-activated receptor alpha
SOCS3: Suppressor of cytokine signaling 3
solTNF: Soluble tumor necrosis factor
SREBP-1C: Sterol regulatory element-binding protein 1c
T2D: Type 2 diabetes
TIRAP: Toll-interleukin 1 receptor (TIR) domain-containing adaptor protein
TlR2: Toll-like receptor 2
TLR4: Toll-like receptor 4
TMTNF: Transmembrane tumor necrosis factor
TNF: Tumor necrosis factor
TNFSf14: Tumor necrosis factor (ligand) superfamily, member 14
ZO-1 (TJP1): Tight junction protein 1
