# Supplementary figures and images for: Targeting soluble tumor necrosis factor as a potential intervention to lower risk for late-onset Alzheimer’s disease associated with obesity, metabolic syndrome, and type 2 diabetes
Source: Alzheimers Res Ther. 2019 Dec 31;12:1. doi: 10.1186/s13195-019-0546-4 (PMC6937979; doi:10.1186/s13195-019-0546-4)

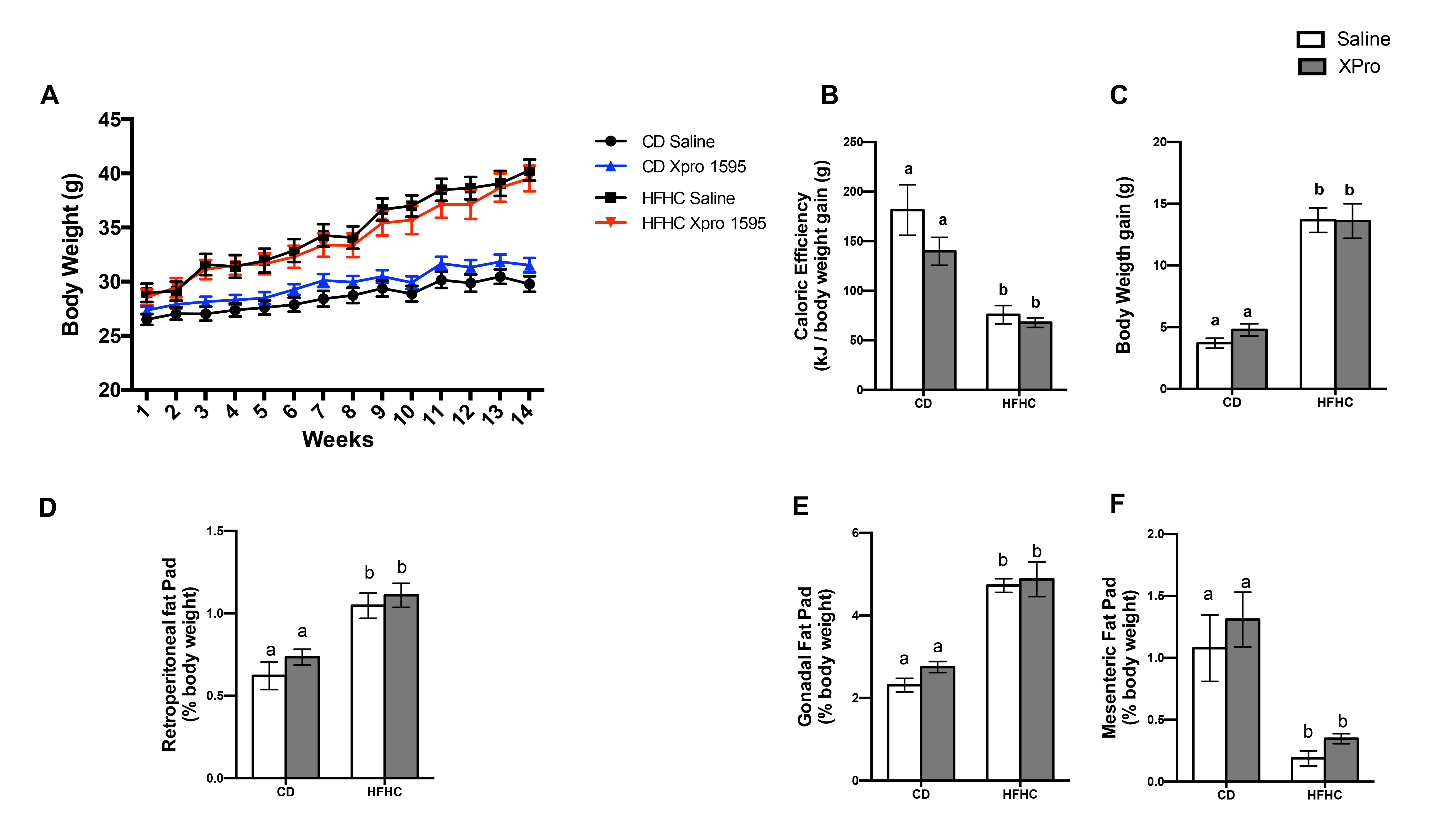

Supplement: Supplementary file 1 — Additional file 1: Figure S1. Impact of HFHC diet consumption on mouse body weight and adiposity. A-F, HFHC diet consumption impacts body-weight gain and caloric efficiency, as well as retroperitoneal, gonadal and mesenteric fat pads. Mice were weighted once a week. Caloric efficiency was obtained by dividing caloric intake (kJ) by changes in body weight. Data were analyzed by two-way ANOVA followed by Tukey’s multiple comparisons in GraphPad Prism 6. Bar height indicates mean of samples from 12-13 mice, error bars indicate standard error of the mean (SEM). Letters indicate post hoc analysis. Means with different letters are significantly different from each other, P <0.05. [file 13195_2019_546_MOESM1_ESM.tif]

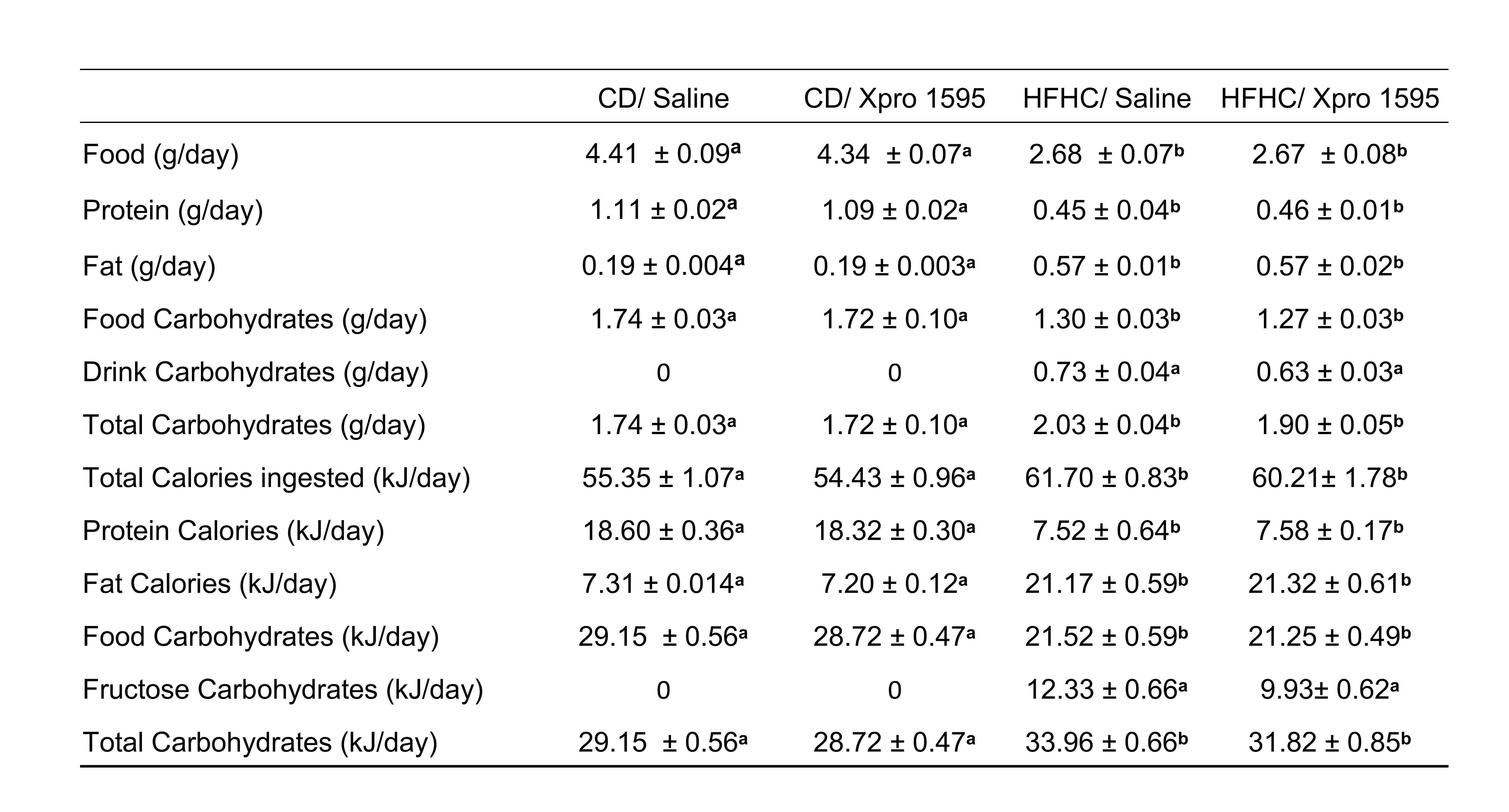

Supplement: Supplementary file 2 — Additional file 2: Table S1. Diet intake*. Values are presented as an average ± s.e.m. Data were analyzed by two-way ANOVA followed by Tukey’s multiple comparisons in GraphPad Prism 6. Letters indicate post hoc analysis. Means with different letters are significantly different from each other, P <0.05, n=12-13 mice. Food and drink intake were measured daily for 10 weeks (food was not measured during behavioral assessment). [file 13195_2019_546_MOESM2_ESM.tif]

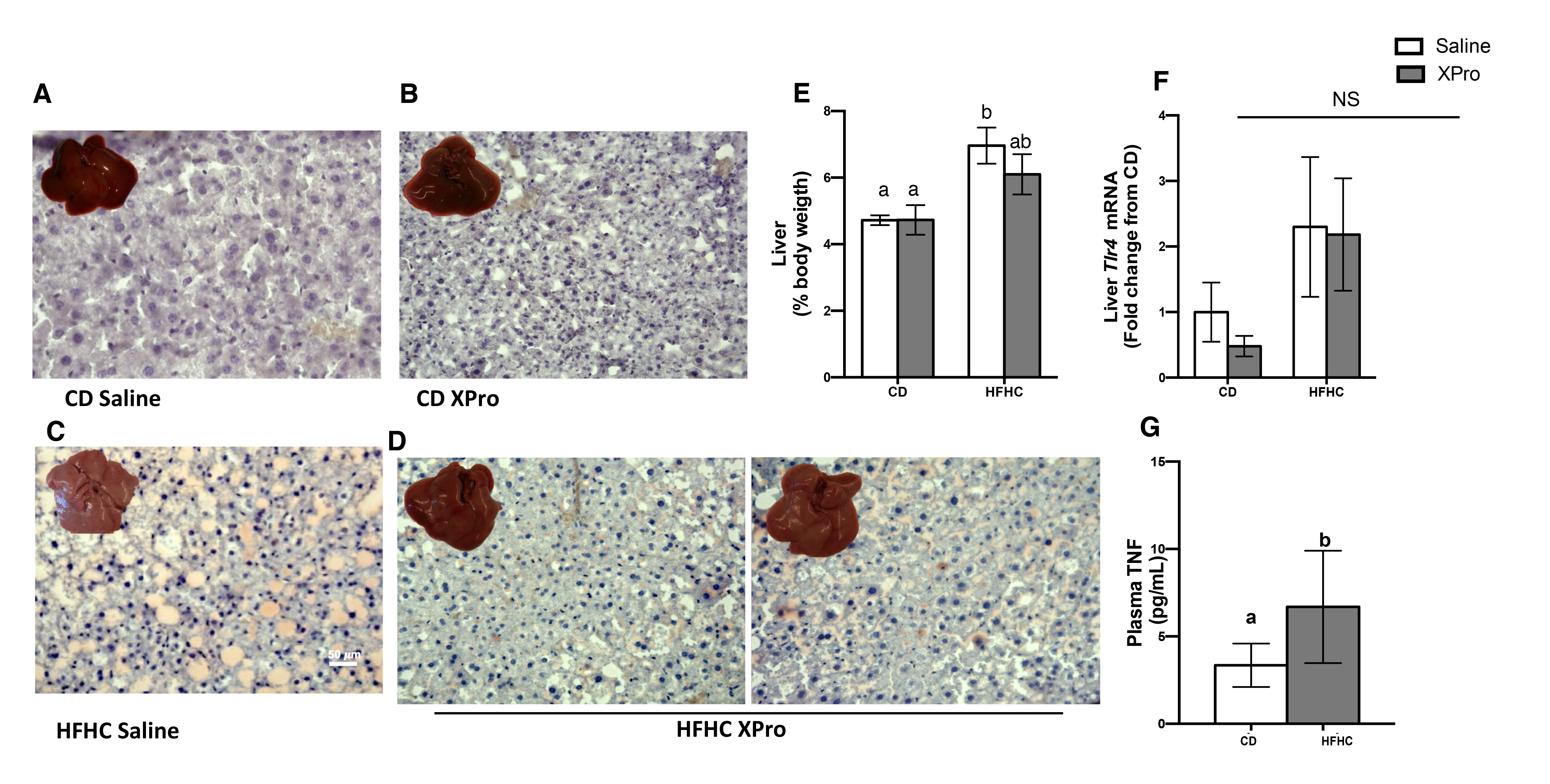

Supplement: Supplementary file 3 — Additional file 3: Figure S2. HFHC diet increases hepatic lipid deposition and liver weight. A-D, Macroscopic appearance and histological sections of representative livers stained for Oil Red O show hepatic lipid accumulation associated with HFHC. E, Increased liver weight (n=12-13) and F, impact on Tlr4 mRNA expression was observed in HFHC groups (n=6). G, HFHC diet increases plasma TNF levels, (n=9-10). Oil Red O/Hematoxylin counterstained liver sections, magnification 40X. Images were obtained using a Nikon Eclipse 90i microscope with a DS-Fi1 (Nikon) camera and Nikon NIS-Elements AR 3.10 software (n=3 per group). Liver tissue was analyzed by qPCR using primers directed against murine Tlr4. For each animal, the Ct values were normalized to the Ct values for Gapdh and Ppia. The relative expression level of the target gene (fold change) was expressed as 2-ΔΔCt, when compared with the mean DCt (threshold cycle) of the CD group. Data were analyzed by two-way ANOVA followed by Tukey’s multiple comparisons and unpaired, two-tailed t-test in GraphPad Prism 6. Data in bar graphs are represented as the mean ± standard error of the mean (s.e.m). Letters indicate post hoc analysis. Means with different letters are significantly different from each other, P<0.05. Note that because of the mechanism of action of XPro1595 (i.e. sequestration of solTNF via formation of heterotrimers), it is not possible to accurately measure the effect of XPro1595 on levels of endogenous solTNF after administration of XPro1595 because the anti-mouse TNF immunoassay captures mouse solTNF homotrimers and mouse heterotrimers with XPro1595 alike). [file 13195_2019_546_MOESM3_ESM.tif]

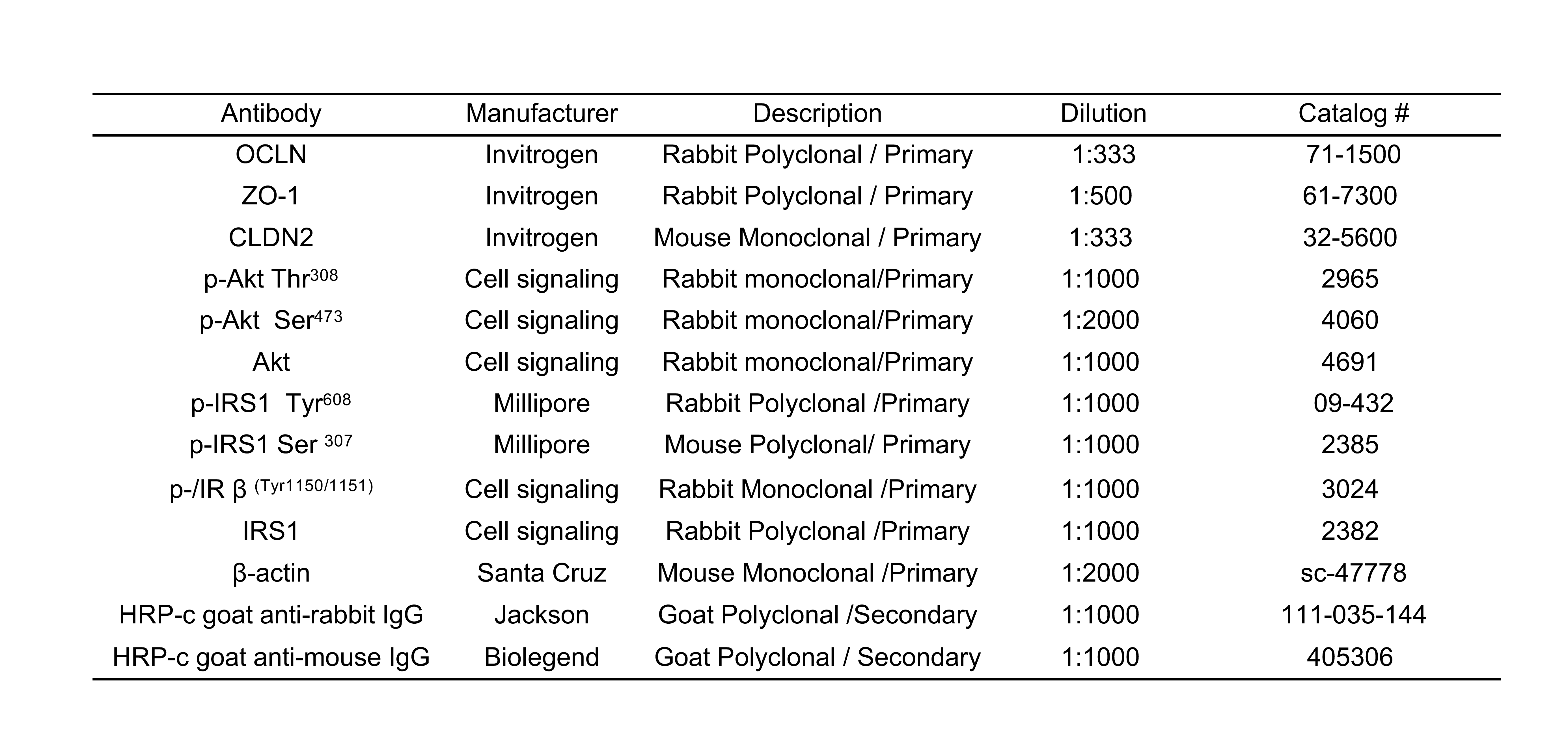

Supplement: Supplementary file 4 — Additional file 4: Table S2. Western blot antibodies. [file 13195_2019_546_MOESM4_ESM.tif]

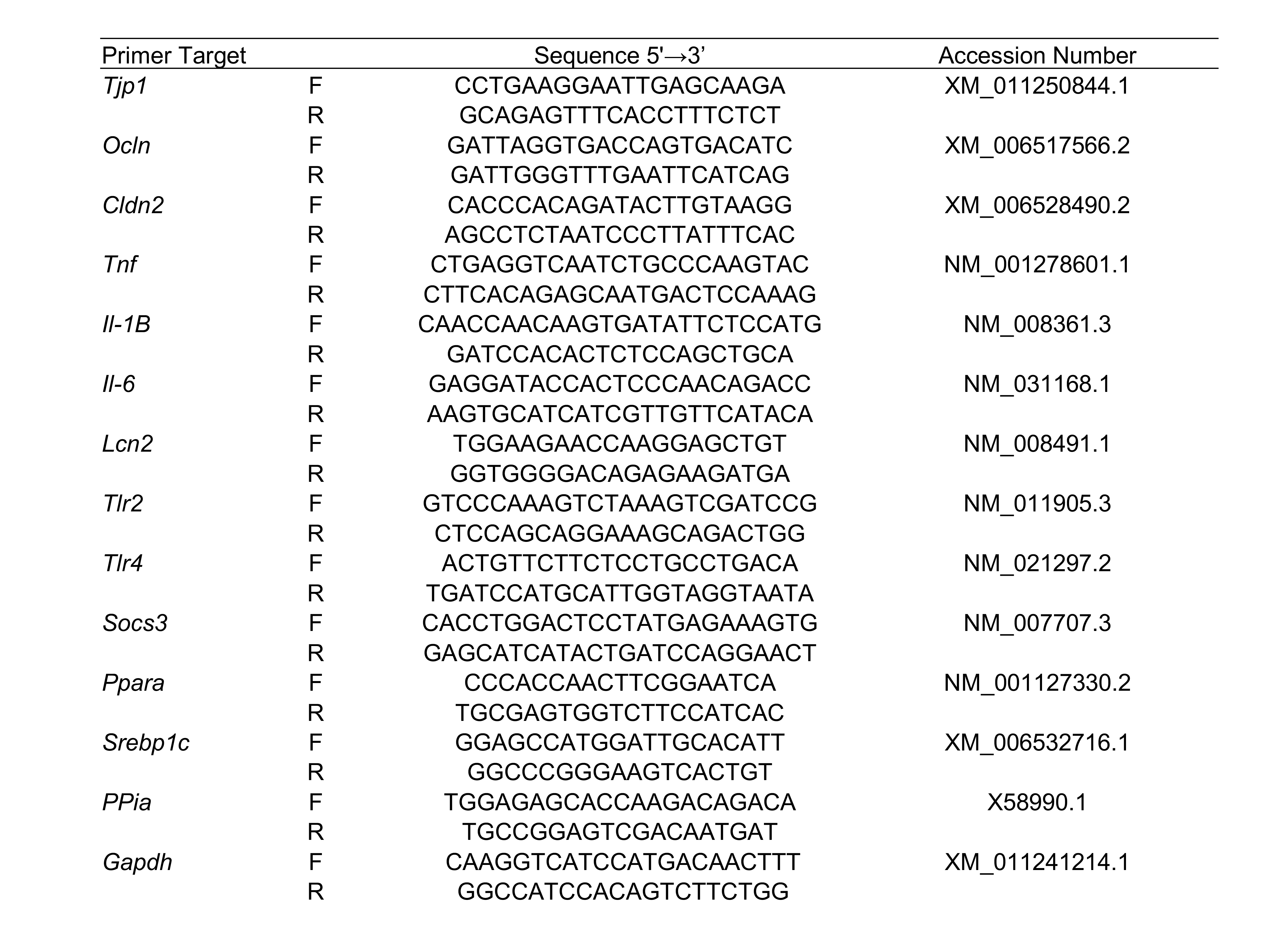

Supplement: Supplementary file 5 — Additional file 5: Table S3. Real time PCR oligonucleotide sequences*. Specific gene sequences were obtained from Genbank at the National Center for Biotechnology Information (http://www.ncbi.nlm.nih.gov/). Primer specificity was verified using the Basic Local Alignment Search Tool (http://www.ncbi.nlm.nih.gov/blast/). Tight junction protein 1 (Tjp1), Occludin (Ocln), Claudin-2 (Cldn2), Tumor necrosis factor (Tnf); Interleukin 1B and 6 (Il-1B) and (Il-6); Lipocalin-2 (Lcn2); Toll-like receptor 2 (Tlr2), Toll-like receptor 4 (Tlr4), Suppressor of cytokine signaling 1 and 3 (Socs1 and Socs3), Peroxisome proliferator-activated receptor gamma (Ppara), Sterol regulatory element-binding protein 1c (Srebp-1c), Glyceraldehyde-3-Phosphate Dehydrogenase (Gapdh) and cyclophilin E (Cyclo). [file 13195_2019_546_MOESM5_ESM.tif]
